# Wnt Signaling Regulates the Lineage Differentiation Potential of Mouse Embryonic Stem Cells through Tcf3 Down-Regulation

**DOI:** 10.1371/journal.pgen.1003424

**Published:** 2013-05-02

**Authors:** Yaser Atlasi, Rubina Noori, Claudia Gaspar, Patrick Franken, Andrea Sacchetti, Haleh Rafati, Tokameh Mahmoudi, Charles Decraene, George A. Calin, Bradley J. Merrill, Riccardo Fodde

**Affiliations:** 1 Department of Pathology, Josephine Nefkens Institute, Erasmus MC, Rotterdam, The Netherlands; 2 Department of Biochemistry, Erasmus MC, Rotterdam, The Netherlands; 3 Translational Research Department, Institut Curie, Centre de Recherche, Paris, France; 4 CNRS, UMR144, Paris, France; 5 Department of Experimental Therapeutics and Center for RNA Interference and Non-Coding RNAs, MD Anderson Cancer Center, Houston, Texas, United States of America; 6 Department of Biochemistry and Molecular Genetics, University of Illinois, Chicago, Illinois, United States of America; University of Michigan, United States of America

## Abstract

Canonical Wnt signaling plays a rate-limiting role in regulating self-renewal and differentiation in mouse embryonic stem cells (ESCs). We have previously shown that mutation in the *Apc* (adenomatous polyposis coli) tumor suppressor gene constitutively activates Wnt signaling in ESCs and inhibits their capacity to differentiate towards ecto-, meso-, and endodermal lineages. However, the underlying molecular and cellular mechanisms through which Wnt regulates lineage differentiation in mouse ESCs remain to date largely unknown. To this aim, we have derived and studied the gene expression profiles of several *Apc*-mutant ESC lines encoding for different levels of Wnt signaling activation. We found that down-regulation of *Tcf3*, a member of the Tcf/Lef family and a key player in the control of self-renewal and pluripotency, represents a specific and primary response to Wnt activation in ESCs. Accordingly, rescuing Tcf3 expression partially restored the neural defects observed in *Apc*-mutant ESCs, suggesting that *Tcf3* down-regulation is a necessary step towards Wnt-mediated suppression of neural differentiation. We found that *Tcf3* down-regulation in the context of constitutively active Wnt signaling does not result from promoter DNA methylation but is likely to be caused by a plethora of mechanisms at both the RNA and protein level as shown by the observed decrease in activating histone marks (H3K4me3 and H3-acetylation) and the upregulation of miR-211, a novel Wnt-regulated microRNA that targets Tcf3 and attenuates early neural differentiation in mouse ESCs. Our data show for the first time that Wnt signaling down-regulates *Tcf3* expression, possibly at both the transcriptional and post-transcriptional levels, and thus highlight a novel mechanism through which Wnt signaling inhibits neuro-ectodermal lineage differentiation in mouse embryonic stem cells.

## Author Summary

The future successes of regenerative medicine largely rely on our knowledge of, and our capacity to manipulate, the cellular and molecular mechanisms governing stem cell differentiation. A growing body of evidence suggests that, in mouse embryonic stem cells, canonical Wnt/β-catenin signaling not only enhances self-renewal but also directs the cell fate decision towards non-neuroectodermal lineages. However, little is known about the mechanisms underlying the differentiation defects caused by constitutive active Wnt signaling. Using a set of *Apc*-mutant ESCs harbouring different levels of Wnt signaling, we found that, among others, down-regulation of *Tcf3*, a key member of the pluripotency circuit, as well as induction of a novel Wnt-regulated microRNA, miR-211, represent two important downstream effects through which Wnt signaling inhibits neural differentiation in mouse ESCs. We also provide a more detailed picture on how Wnt signaling counteracts Tcf3 function in stem cells by showing that Tcf3 repression, in the context of active Wnt signaling, involves histone modifications at the *Tcf3* promoter and the activation of miR-211, which post-transcriptionally stabilizes Tcf3 downregulation. Understanding the downstream effects of Wnt signaling in ESCs is of both fundamental and translational relevance, as it may be exploited to manipulate ESC differentiation towards specific cell lineages.

## Introduction

Embryonic stem cells (ESCs) are *in vitro* cultured cells derived from the preimplantation-stage embryo, which possess unconfined capacity for self-renewal and multi-lineage differentiation towards different embryonic germ layers. Pluripotency and self-renewal are two essential features of ESCs, which make them not only a very robust and suitable model for stem cell research, but also a promising source for regenerative medicine. Also, with the emergence of induced pluripotent stem cells (iPS) technology, understanding the basic mechanisms governing the embryonic stem state becomes of great interest for safe clinical applications in regenerative medicine and stem cell programming.

Among different signaling pathways, Wnt/β-catenin signaling has been shown to play a major role in maintaining self-renewal as well as in regulating ESCs differentiation [1], [2], [3],[4],[5],[6]. The canonical Wnt/β-catenin signaling pathway is controlled by post-translational modifications of β-catenin leading to its differential protein stability and sub-cellular localization. In the absence of active Wnt signaling, β-catenin is negatively regulated by the so-called “destruction complex”, consisting of the Apc and Axin scaffolding proteins and the glycogen synthase and casein kinases (GSK and CK1), resulting in proteolytic degradation and low levels of cytoplasmic β-catenin. Ligand-mediated Wnt signaling activation leads to nuclear translocation of β-catenin where it binds to members of the Tcf/Lef family of transcriptional factors thus modulating the expression of a broad spectrum of downstream target genes [7], [8], [9].

In vertebrates, the Tcf/Lef family encompasses four functionally specialized members including Tcf1 (also known as Tcf7), Tcf3 (also known as Tcf7l1), Tcf4 (also known as Tcf7l2) and Lef1 [10]. Whereas Tcf1, Tcf4 and Lef1 are known to activate different Wnt target genes in the context of active Wnt signaling, Tcf3 primarily functions as a transcriptional repressor [5], [11], [12], [13], [14], [15], [16]. Tcf3 is the most abundant Tcf/Lef member in mouse ES cells [14] and is an integral component of the core pluripotency circuit, co-occupying Oct4, Nanog and Sox2 DNA binding sites [17], [18], [19], [20]. Loss of function experiments have shown that Tcf3 down-regulation enhances self-renewal and confers differentiation resistance in mouse ESCs [14], [17], [19], [20], [21], [22]. In fact, both the zebrafish *headless/tcf3* mutant and the *Xenopus* embryo depleted of TCF3 reveal anterior head defects resembling the Wnt-gain of function phenotype [11], [15], [16]. Similarly, Tcf3 ablation in mice resulted in expanded axial mesoderm and loss of anterior neural tissues [21]. *Tcf3* is ubiquitously expressed through the mouse embryo at embryonic day 6.5 (E6.5) and is gradually localized in the anterior part of the embryo at E7.5 and the anterior neuroectoderm at E8.5 [23], [24].

Although several studies have demonstrated the key role played by Wnt signaling in regulating self-renewal and differentiation of both mouse and human ESCs, the downstream effects through which Wnt exerts these functions have been a matter of controversy. To date, three models have been suggested in this regard: a. Tcf-independent, β-catenin/Oct4 signaling [25]; b. Tcf3 antagonism by nuclear β-catenin which relieves Tcf3 repression and enhances self-renewal. A minimal role for the canonical Tcf/β-catenin signaling has been suggested in this model [6]; and c. synergistic action of Tcf3 antagonism and the canonical β-catenin/Tcf1 signaling [5]. Although these studies have shed some light on the underlying mechanisms through which Wnt signaling controls self-renewal, none of the above-mentioned models explains how this signaling pathway regulates the lineage differentiation potential of ESCs.

In order to elucidate the downstream effects of Wnt signaling on lineage commitment and differentiation in embryonic stem cells, we examined several *Apc*-mutant ESCs harboring different levels of Wnt signaling and compared their gene expression profiles with wild type ESCs. We show that activation of Wnt signaling down-regulates Tcf3 expression in mouse ESCs. We provide evidence that *Tcf3* down-regulation represents a main downstream effect through which Wnt signaling directs the differentiation of pluripotent ESCs towards non-neuroectodermal lineages. Moreover, we show that Wnt-mediated repression of *Tcf3* involves epigenetic regulation associated with histone modifications and Wnt-mediated induction of miR-211. Our data demonstrate that Wnt signaling counteracts Tcf3 function at multiple levels, which ultimately ensures the delicate balance between self-renewal and differentiation in mouse ESCs.

## Results

### Lineage differentiation in *Apc*-mutant ESCs correlates with the level of Wnt signaling

**Figure 1:**
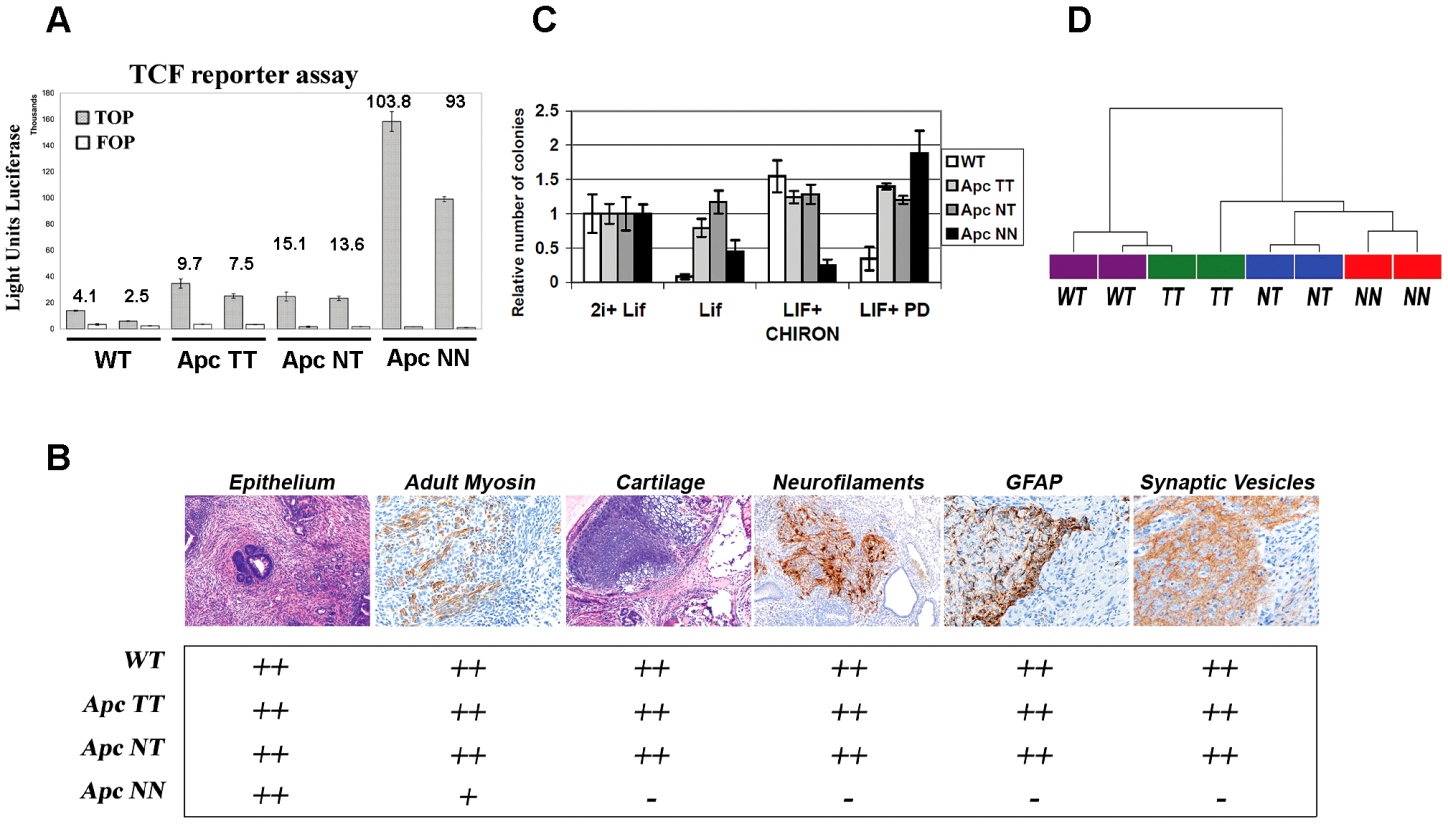
Wnt signaling regulates the differentiation potential of mouse ESCs in a dosage-dependent manner. A. β-catenin/TCF reporter assay in wild type and *Apc*-mutant ESCs. Measurements are reported as the average luciferase units performed in triplicate for the TOP (filled bars) and FOP (empty bars) reporter constructs (data reported is mean±SD). Numbers in the histogram represent the calculated TOP/FOP ratios. B. Table summarizing the results obtained by teratoma differentiation assay from different *Apc*-mutant ESCs and their wild type controls. Tissue sections were stained with hematoxylin and eosin (H&E) or used in immunohistochemical analysis using specific antibodies against the neural markers: GFAP, neurofilaments and synaptic vesicles. Adult myosin was used as a mesodermal marker to stain the striated muscle differentiation. Cartilage differentiation was assessed either by H&E or theonin staining. Two independent clones were used for each genotype and differentiation was scored as: (−) not present, (+) weakly present, and (++) present. C. Histogram showing the percent of colonies formed after plating 500 FACS-sorted cells in N2B27 medium supplemented with different combinations of LIF, Mek inhibitor (PD) and GSK-inhibitor (CHIRON). Bars represent mean ± SD, n = 3. D. Dendrogram derived from unsupervised hierarchical clustering of global gene expression in wild type, *Apc*TT, *Apc*NT and *Apc*NN ES cells. Pearson's correlation coefficient and Ward's method were used after MAS 5.0 normalization of all probe sets.

To attempt the elucidation of the mechanisms underlying lineage differentiation in the context of Wnt activation, we have derived several ES clones from pre-implantation blastocysts carrying different hypomorphic *Apc* alleles: *Apc* ^1638T/1638T^ (*Apc*TT), *Apc* ^1638N/1638T^ (*Apc*NT), *Apc* ^1638N/1638N^ (*Apc*NN) [26], [27], together with *Apc* ^+/+^ as wild type controls. As previously reported, *Apc*TT, *Apc*NT, and *Apc*NN encode for a gradient of different Wnt signaling dosages [1], [26], as also confirmed by TOP-Flash reporter assay [28] with *Apc*NN showing the highest Wnt activity (*Apc*NN≫*Apc*NT>*Apc*TT>*Apc* ^+/+^)(Figure 1A). The potential of the *Apc*-mutant ES cells to differentiate into ecto-, meso- and endodermal lineages was also evaluated and confirmed by the teratoma formation assay followed by immunohistochemistry (IHC) analysis, matching our previous results obtained with ES clones obtained by two rounds of gene targeting by homologous recombination [1]. As expected, no expression of neuroectodermal markers (GFAP, SV2, and neurofilaments) was observed in teratomas derived from *Apc*NN ES cells (Figure 1B).

ES cells can be cultured in serum-free medium supplemented with LIF, GSK inhibitor (CHIRON) and Mek inhibitor (PD), the so-called 2i medium [29]. Using the serum-free culture supplemented with a single inhibitor, we found that *Apc*NN cells have the highest colony-forming capacity when cultured in LIF+Mek inhibitor, suggesting that their constitutive Wnt signaling activity replaces the need for additional pathway activation by the GSK inhibitor (Figure 1C). Of note, culturing *Apc*NN ESCs in medium supplemented with CHIRON reduced the colony formation capacity of these cells suggesting that a very high dosage of Wnt signaling can compromise the growth of *Apc*NN cells. We also observed that *Apc*TT and *Apc*NT cells formed similar number of colonies in different culture conditions independently of CHIRON supplementation, possibly pointing to the Wnt-independent effects of *Apc* mutations in these cells (Figure 1C).

### Wnt signaling down-regulates *Tcf3* expression in mouse ESCs

**Figure 2:**
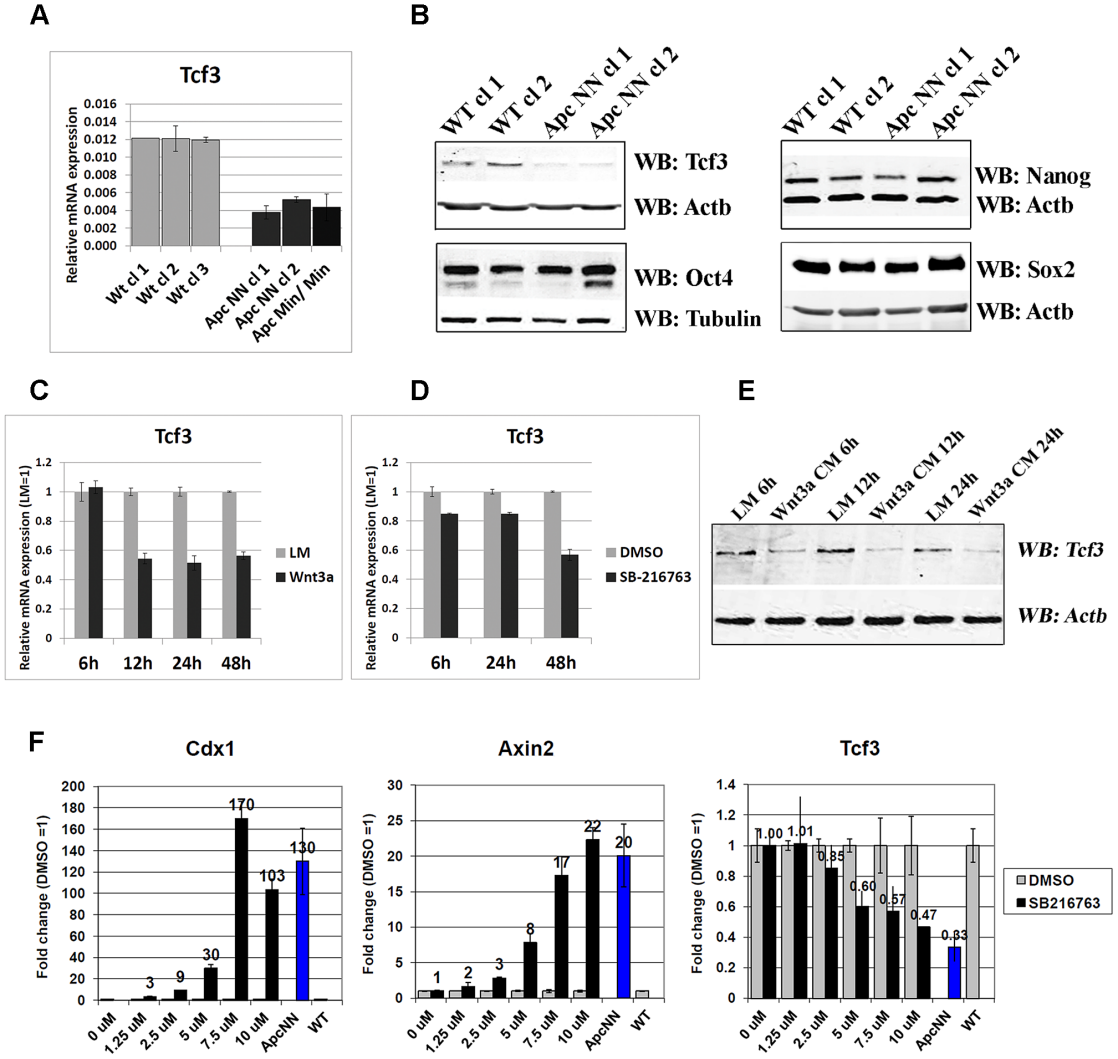
Wnt signaling downregulates Tcf3 expression in mouse ESCs. A. qRT-PCR analysis of *Tcf3* in wild type, *Apc*NN and *Apc* ^Min/Min^ ESCs. *Actb* was used as an internal control; bars represent n = 2 ± SD. B. Western blot analysis of the core pluripotency markers Oct4, Nanog, Sox2 and Tcf3 on protein lysates isolated from two independent *Apc*NN clones and wild type control ESCs. Actb and Tubulin were used as an internal control. C–D. qRT-PCR analysis of *Tcf3* in wild type ESCs treated for different time intervals with Wnt3a conditioned medium (C), or with the GSK-inhibitor SB-216763 (D). L-medium and DMSO were employed as controls, respectively. *Actb* was used as an internal control; bars represent n = 2 ± SD. E. Time course western blot analysis of Tcf3 expression in wild type ESCs treated with Wnt3a conditioned medium (Wnt3a CM) or control L-medium (LM). *Actb* was used as an internal control. F. qRT-PCR analysis of *Tcf3* and Wnt target genes Axin2 and Cdx1 in wild type ESC treated for 48 h with different concentrations of GSK-inhibitor SB-216763 or DMSO as control. *Actb* was used as an internal control; bars represent n = 2 ± SD.

To elucidate the molecular mechanisms underlying the altered cell fate decision in *Apc*-mutant ES cells, genome-wide transcriptional analysis was performed on the newly derived cells. Unsupervised hierarchical clustering analysis showed that global gene expression in *Apc*NN ESCs is already influenced before differentiation is induced, resolving *Apc*NN from WT expression profiles in different branches of the dendogram (Figure 1D). Among the genes differentially expressed between *Apc*NN ES cells and their wild type counterparts (Table S1), we found that, unlike other pluripotency markers (e.g. *Oct4*, *Nanog*, and *Sox2*), *Tcf3* was specifically down-regulated in *Apc*NN ES cells, an observation which was further confirmed by qRT-PCR and western blot analysis (Figure 2A and 2B; and Figure S1). Further qRT-PCR analysis revealed that the observed downregulation is specific for *Tcf3* but not for other members of the Tcf/Lef family (Figure S2A). Whereas Tcf3 was down-regulated in both *Apc*NN and *Apc* ^Min/Min^ ESCs, the latter encode for the most severely truncated Apc mutant allele and therefore for a very high level of Wnt signaling, other members of the Tcf/Lef family were exclusively up-regulated in *Apc* ^Min/Min^ ESCs.

Accordingly, Wnt activation achieved in wild type cells either by Wnt3a conditioned medium or by a GSK3-small molecule inhibitor (SB-216763), confirmed that *Tcf3* down-regulation is a specific response to canonical Wnt signaling in mouse ESCs (Figure 2C, 2D, and 2E, and Figure S2B and S2C). Moreover, using a gradient of the GSK inhibitor SB-216763, we observed that unlike the canonical Wnt targets *Axin2* and *Cdx1*, downregulation of *Tcf3* required a higher Wnt signaling level, possibly explaining why *Tcf3* downregulation is observed in *Apc*NN cells but not *Apc*TT or *Apc*NT ESCs (Figure 2F and Figure S1).

### Rescuing *Tcf3* expression in *Apc*NN ESCs partially restores neural differentiation

**Figure 3:**
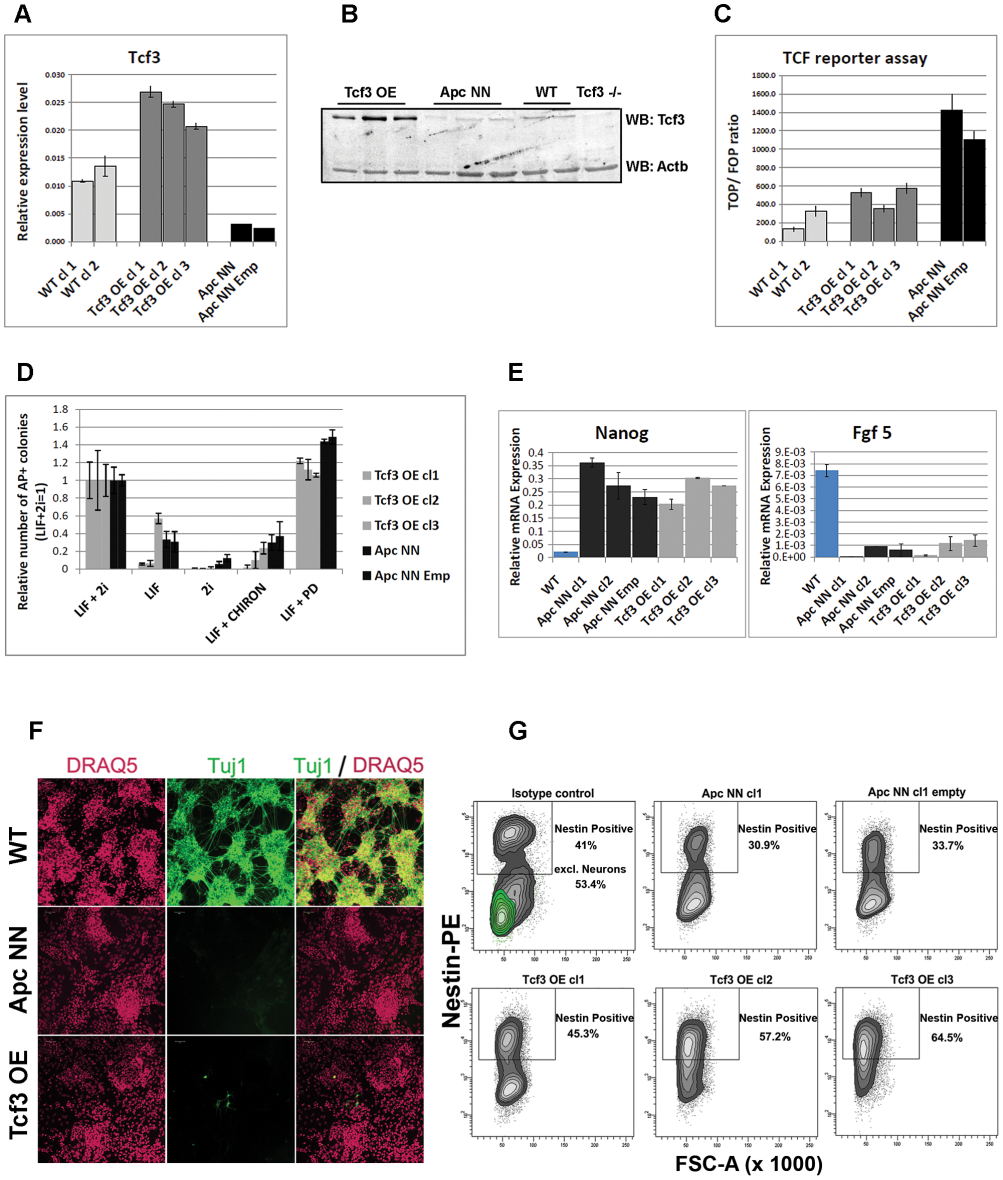
Characterization of Tcf3 over expressing ESCs. A–B. qRT-PCR (A) and western blot (B) analysis of Tcf3 in *Apc*NN ESCs stably expressing Tcf3. Wild type and *Tcf3* ^−/−^ ESCs were used for comparison. Actb was used as an internal control. C. Histogram showing reduction of β-catenin/Tcf reporter activity in *Apc*NN cells stably expressing Tcf3 (Tcf3 OE) compared to parental *Apc*NN cells and cells expressing the corresponding empty vector. Luciferase signal from TOP or FOP reporter constructs were measured and TOP/FOP ratios are shown in the graph. Bars represent n = 3 ± SD. D. Histogram showing the percent of alkaline phosphatase (AP) positive colonies formed by plating 500 FACS-sorted cells in N2B27 medium after 7 days. N2B27 medium was supplemented with different combinations of LIF, PD and CHIRON. Two independent *Apc*NN ESC clones (parental clone and transfected with empty vector) and three independent *Apc*NN ESC clones expressing *Tcf3* (Tcf3 OE) were used. Bars represent n = 3 ± SD. E. Histograms showing relative expression of the pluripotency markers *Nanog* and the early differentiation markers *Fgf5* in different ESCs cultured for 48 h in N2B27 medium. F. Confocal analysis of ES cells stained with Tuj-1-Alexa 488 and counterstained with the far-red nuclear stain DRAQ5. Wild type, *Apc*NN and *Apc*NN expressing *Tcf3* (Tcf3 OE) ESCs were used in −4/+4 neural differentiation assay and analyzed by immunofluorescence after 13 days of culture. G. Flow cytometric analysis showing expression of the neural progenitor marker Nestin in *Apc*NN ESCs stably expressing *Tcf3* (Tcf3 OE) and their control cells (parental *Apc*NN clone and *Apc*NN transfected with the corresponding empty vector) or wild type ESCs. Cells were analyzed by the −4/+4 neural differentiation assay and stained with specific antibody against Nestin and Tuj1 after 13 days of culture. Wild type (WT) ESCs are shown as control to indicate the Tuj1 positive population which is absent in other genotypes (0.1% in average in Tcf3 OE clones). Numbers in the graph represent the percent of Nestin-positive cells. For wild type ESCs the Nestin-positive populations before and after excluding the mature neurons are shown. See also Figure S4 for defining different FACS gates.

It has been previously shown that *Tcf3* not only functions as a controller of self-renewal in wild type ESCs, but it is also required for proper neurogenesis in zebrafish, xenopus and mice [11], [16], [21]. We therefore hypothesized that *Tcf3* down-regulation in *Apc*NN ESCs might mediate the neural differentiation defects observed in these cells. To test this hypothesis, we rescued *Tcf3* expression by stably over-expressing its full-length cDNA in *Apc*NN ES cells (Figure 3A, 3B). *Tcf3* over-expression decreased TOP-Flash reporter activity (Figure 3C) and, accordingly, reduced the transcript levels of *Cdx1* and *Brachyury* (*T*), two well-known Wnt downstream targets. Gene expression profiling of *Tcf3*-expressing *Apc*NN cells confirmed that *Tcf3* effectively reverses the expression pattern of several genes differentially expressed in *Apc*NN when compared to wild type ESCs (Figure S3).

Since it has been previously reported that Tcf3 over-expression in wild type ESCs induces differentiation under self-renewing conditions [5], we first assessed whether over expressing Tcf3 in *Apc*NN ESCs induces similar effects in these cells. As reported above, *Apc*NN cells can grow in 1i medium (i.e. in LIF+Mek inhibitor) in the absence of GSK inhibitor (Figure 1C). To investigate whether Tcf3 can restore their dependency on the GSK inhibitor in serum-free culture, *Tcf3*-over expressing *Apc*NN cells were seeded at clonal density under different conditions and subsequently stained for alkaline phosphatase (AP) to evaluate the percentage of undifferentiated colonies. We found that, similar to the parental *Apc*NN cells, *Tcf3*-rescued clones show the highest colony forming capacity in the presence of LIF and Mek inhibitor (Figure 3D). Moreover, by applying the short term differentiation assay in N2B27 medium, we found that both *Apc*NN and their *Tcf3*-rescued counterparts retain expression of the pluripotency markers *Nanog* while fail to express the early differentiation markers *Fgf5* (Figure 3E). Hence, constitutive Wnt signaling prevents differentiation in a short-term assay despite the ectopic *Tcf3* expression.

**Figure 4:**
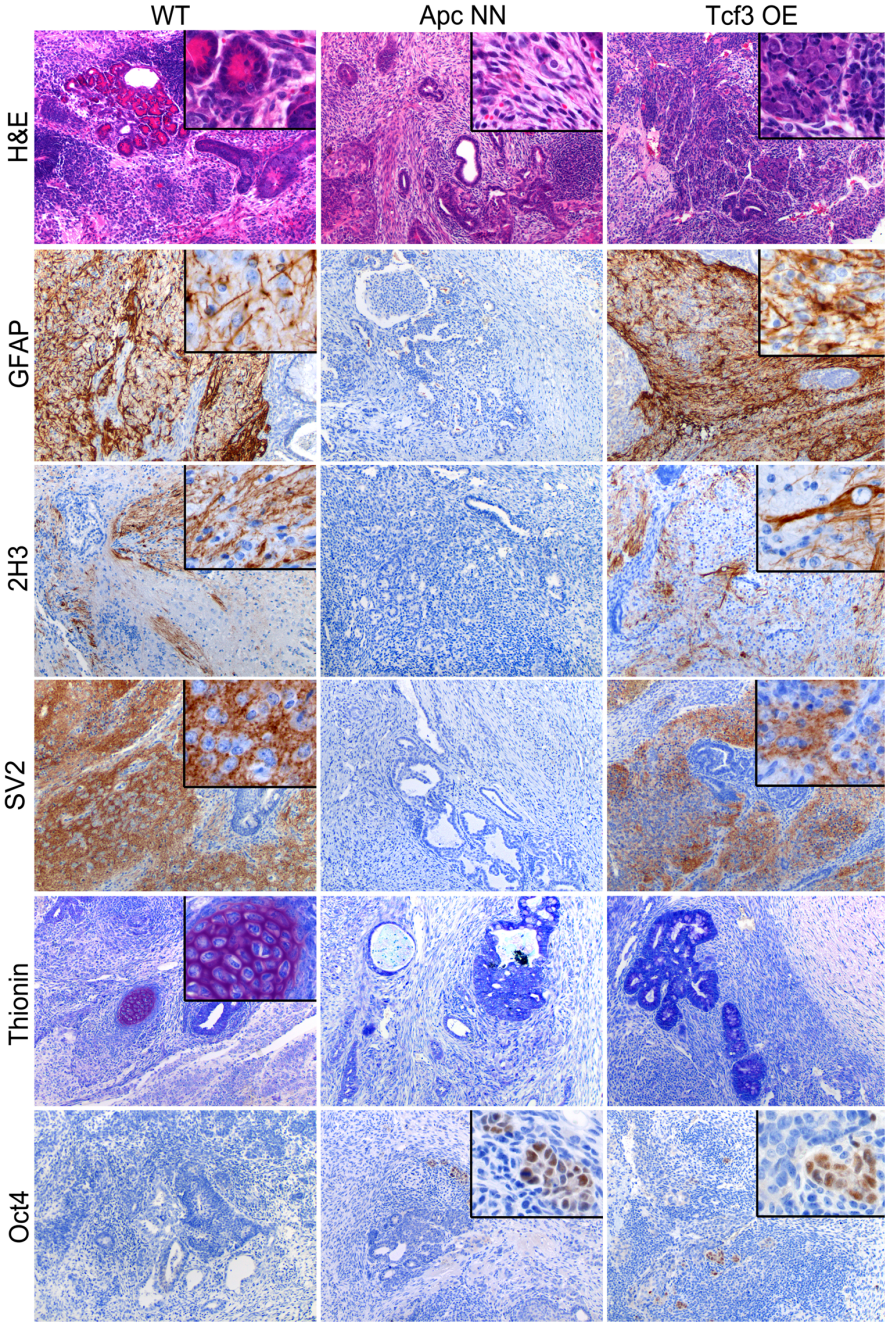
Rescue of *Tcf3* expression in *Apc*NN ESCs partially restores *in vivo* neural differentiation. Teratoma samples were obtained from wild type, *Apc*NN and *Apc*NN stably expressing *Tcf3* (Tcf3 OE) ESCs. Tissue sections were stained by H&E, thionin (marker of cartilage differentiation), and by IHC with specific antibodies against the neural differentiation markers GFAP, 2H3 (neurofilaments) and SV2 (synaptic vesicles). Oct4 IHC analysis was used to asses the presence of undifferentiated EC-like cells in the teratomas.

We then asked whether rescuing Tcf3 expression in *Apc*NN cells could affect the neural differentiation potential of these cells. To this aim, we applied the *in vitro* neural differentiation assay previously described by Bibel et al. [30]. We found that, whereas wild type ESCs readily gave rise to Tuj1-positive cells, no staining could be detected in *Apc*NN cells, while only few dispersed Tuj1-expressing cells were observed in the *Tcf3* rescued clones (Figure 3F). In contrast, a clear increase in *Nestin* expression was observed in *Tcf3* over-expressing cells (Figure 3G and Figure S4). This suggests that, although *Tcf3* could not restore the formation of fully mature Tuj1-proficient neurons, it does affect neural differentiation *in vitro* in a more subtle fashion towards neural progenitor-like cells. Next, we examined the differentiation potential of *Tcf3*-rescued ES cells *in vivo* by teratoma assay. We injected the newly generated clones into recipient isogenic mice to generate teratomas and analyzed them for the expression of different neuroectodermal markers by IHC. Interestingly, in contrast to the control *Apc*NN teratomas which did not express any neuroectodermal marker (0/20 analyzed teratomas), approximately 50% of all teratomas generated from different *Tcf3* over-expressing ES clones were positive for the same set of markers (6/10, 6/10, and 4/10 teratomas originated from clones 1, 2 and 3, respectively)(Figure 4). However, the extent of neural differentiation was lower compared to teratomas originated from wild type ESCs. Unlike neuroectodermal lineages, Tcf3 did not rescue the mesodermal cartilage-differentiation defect.

The observed difference in the results obtained by *in vivo* and *in vitro* differentiation assay might reflect the presence of different microenvironmental factors and the longer period of differentiation *in vivo*, which result in a larger extent of neural differentiation in teratomas.

**Figure 5:**
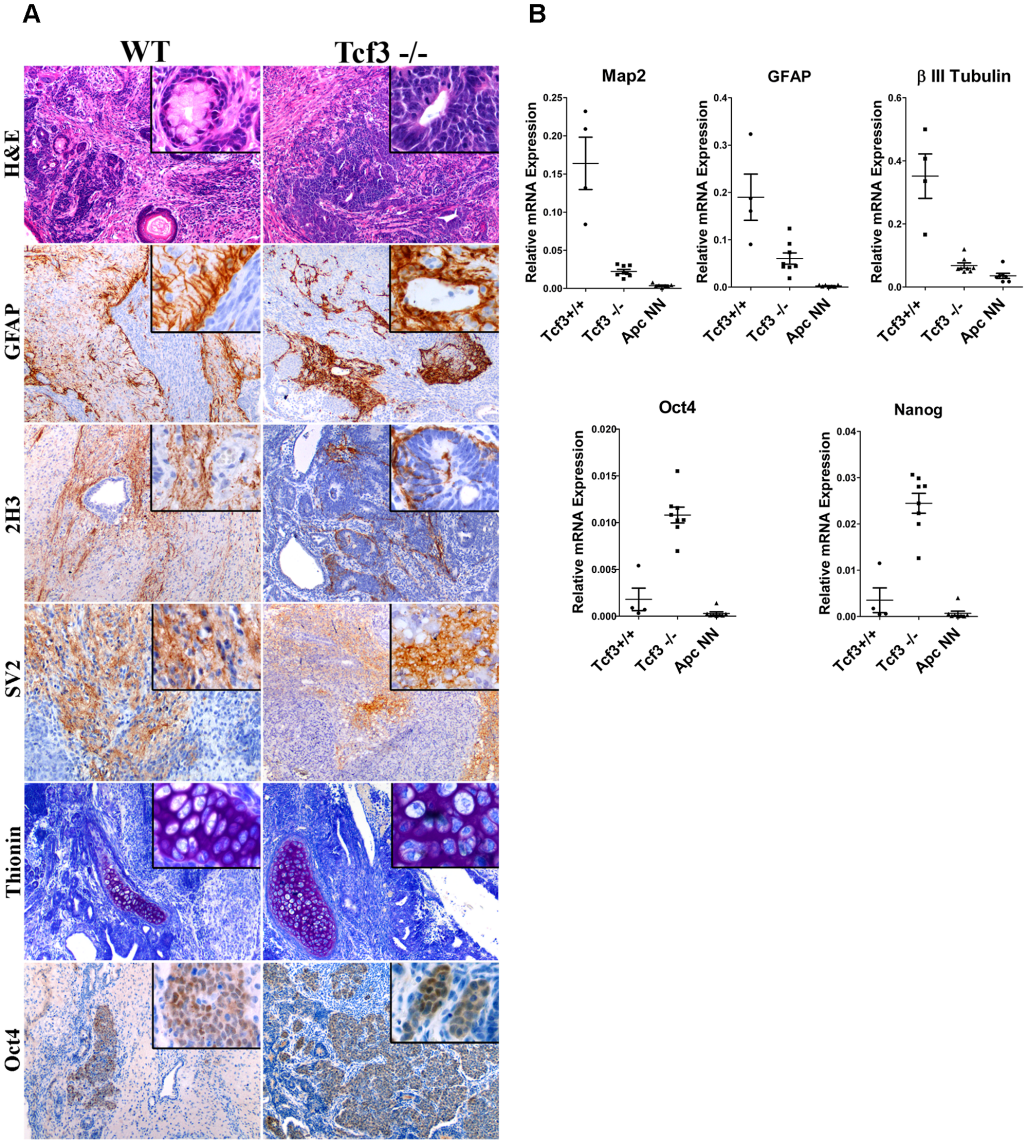
Tcf3 downregulation in wild-type ES cells impairs but does not fully inhibit neural differentiation. A. Immunohistochemistry analysis was used to evaluate the neural differentiation in teratoma samples derived from *Tcf3^−/−^* or their wild type control (GS1) ESCs. Immunostaining with specific antibodies revealed retention of the pluripotency marker Oct4 and expression of the neural markers GFAP, neurofilaments (2H3) and synaptic vesicles (SV2) in *Tcf3^−/−^* teratomas. Thionin staining was used to evaluate cartilage differentiation. B. RNAs were isolated from different teratoma samples and analyzed by qRT-PCR for differentiation markers. Dot plots show normalized qRT-PCR values for the neural markers *Map2*, β-*III-Tubulin* and *GFAP* and for the pluripotency markers *Oct4* and *Nanog* among the different teratoma samples. Each dot represents one sample.

Overall, these results indicate that *Tcf3* expression in *Apc*NN cells can partially rescue the neural differentiation defect characteristic of these cells. Next, we then asked whether Tcf3 down-regulation in wild type embryonic stem cells is sufficient to induce neural differentiation defects, characteristic of Wnt-high ESCs. To this aim, teratomas were obtained by subcutaneous transplantation of *Tcf3* ^−/−^ ESCs [14] followed by IHC and qRT-PCR analysis of different neural markers. We observed reduced neural differentiation in *Tcf3* ^−/−^ teratomas when compared to wild type controls (Figure 5). However, high expression of the pluripotency markers *Oct4* and *Nanog* was also observed in *Tcf3* ^−/−^ teratomas (Figure 5). IHC analysis of Oct4 also showed that *Tcf3* ^−/−^ teratomas are largely composed of undifferentiated, embryonic carcinoma (EC) -like cells, confirming their undifferentiated nature. This is in contrast with *Apc*NN teratomas where pluripotency markers were down-regulated. These results suggest that *Tcf3* down-regulation in wild type ES cells is necessary but insufficient to fully inhibit neural differentiation, and that canonical Wnt signaling is still required for redirecting the differentiation towards non-neuroectodermal lineages.

### Tcf3 down-regulation in *Apc*NN ESCs is associated with histone modifications

To elucidate the mechanisms underlying Wnt-driven repression of *Tcf3* expression, we first analyzed its promoter activity in *Apc*NN and wild type ESCs to localize the responsible regulatory elements. We employed luciferase reporter constructs containing a 6.7 kb genomic fragment upstream of the mouse *Tcf3* ATG translation start site as well as a series of different deletion constructs spanning 4.5 kb, 3.5 kb, 2.2 kb, 1.2 kb and 176 bp fragments of the same region (Figure S5A) [31]. The 4.5 kb fragment was previously shown to resemble endogenous *Tcf3* expression in mouse embryo as well as embryonic derived neural stem cells [31]. To test whether Wnt signaling affects Tcf3 promoter activity, we transfected the different *Tcf3* promoter constructs in *Apc*NN and wild type ESCs. Likewise, transfected wild type ESCs were also treated with Wnt3a conditioned medium or L-control medium to examine *Tcf3* promoter activity. Using both approaches, we found that the Wnt-mediated repression of Tcf3 is not regulated by elements located within the 6.7 kb promoter region (Figure S5A). However, we cannot exclude the possibility that long-range enhancer elements located outside the 6.7 kb promoter region might still contribute to the observed *Tcf3* repression in Wnt context.

The mouse *Tcf3* promoter contains a large CpG island extending over exon 1, 2 and 3. This indicates that DNA methylation may play a role in the regulation of *Tcf3* expression [32]. To test whether the observed *Tcf3* down-regulation in *Apc*NN ESCs results from DNA methylation, we employed the bisulfite-conversion method followed by sequencing and methylation-specific PCR to analyze the *Tcf3* promoter in *Apc*NN cells and compare its methylation pattern to wild type ESCs. As depicted in Figure S5B, we found that similar to wild type ESCs, the *Tcf3* promoter is unmethylated in *Apc*NN cells thus suggesting that DNA methylation is unlikely to represent the mechanism underlying Wnt-driven *Tcf3* down-regulation in mouse ESCs.

**Figure 6:**
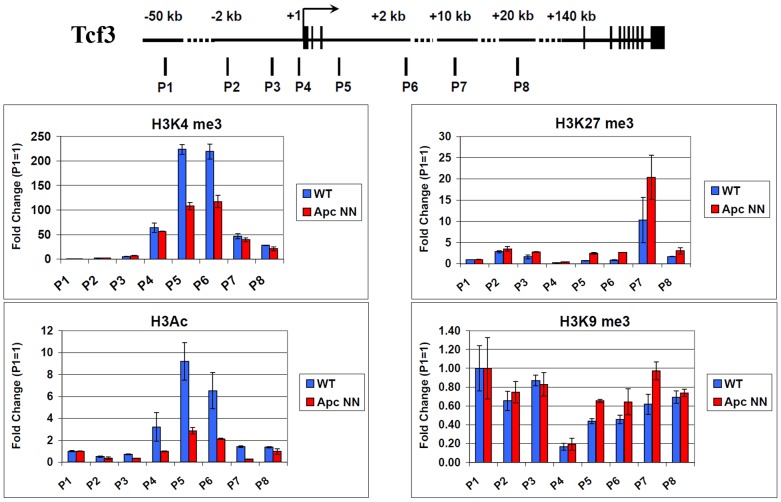
Regulation of Tcf3 in *Apc*NN ESCs is associated with histone modifications. Schematic representation of mouse Tcf3 locus and the different amplicons (P1–P8) analyzed by QPCR in chromatin immunoprecipitation experiment. Chromatin was isolated from *Apc*NN and wild type ESCs and was immunoprecipitated with specific antibodies against the activating histone marks (H3K4me3 and H3Ac) and the repression histone marks (H3k27me3 and H3K9me3). The input DNA (chromatin before immunoprecipitation) and immunoprecipitated DNA was quantified by QPCR and using specific primers as described in materials and methods. Values from each amplicon were normalized to input chromatin and fold change was calculated relative to the corresponding negative region (P1). Bars represent n = 2±SD.

Active and repressed promoters are thought to be associated with histone marks, which reflect the gene expression status of the corresponding genes. To test whether *Tcf3* down-regulation in *Apc*NN cells is regulated via chromatin modifications, we performed chromatin immunoprecipitation (ChIP) to analyze post-translational histone modifications associated with active and repressed promoters. We studied the active-chromatin marks H3K4me3 and H3-acetylation as well as the repressed-chromatin marks H3K27me3 and H3K9me3 in the *Tcf3* promoter and compared the histone modification patterns between *Apc*NN and wild type ESCs. The immunoprecipitated chromatin was then assessed by qPCR analysis with a panel of specific primers covering a region encompassed between −2 kb to +2 kb from the transcription start site (TSS), as well as 20 kb of the gene body within the *Tcf3* locus. In accordance with the observed *Tcf3* downregulation in *Apc*NN cells, we found a decrease in the activating marks H3K4me3 and H3Ac and, to a lesser extent, a slight increase in the repressive marks H3K27me3 and H3K9me3 (Figure 6). Similarly, 12 h treatment of wild type ESCs with Wnt3a conditioned medium significantly reduced the H3Ac and H3K4me3 activating marks but had no effect on the H3K27me3 and H3K9me3 repressing marks (Figure S6). These data demonstrate a correlation between *Tcf3* expression and histone modifications in its promoter suggesting that Wnt signaling might regulate Tcf3 expression through epigenetic mechanisms. However, the mediator of this regulation still remains elusive.

### miR-211, a novel Wnt-regulated microRNA, targets *Tcf3* and attenuates early neural differentiation in wild-type ESCs

**Figure 7:**
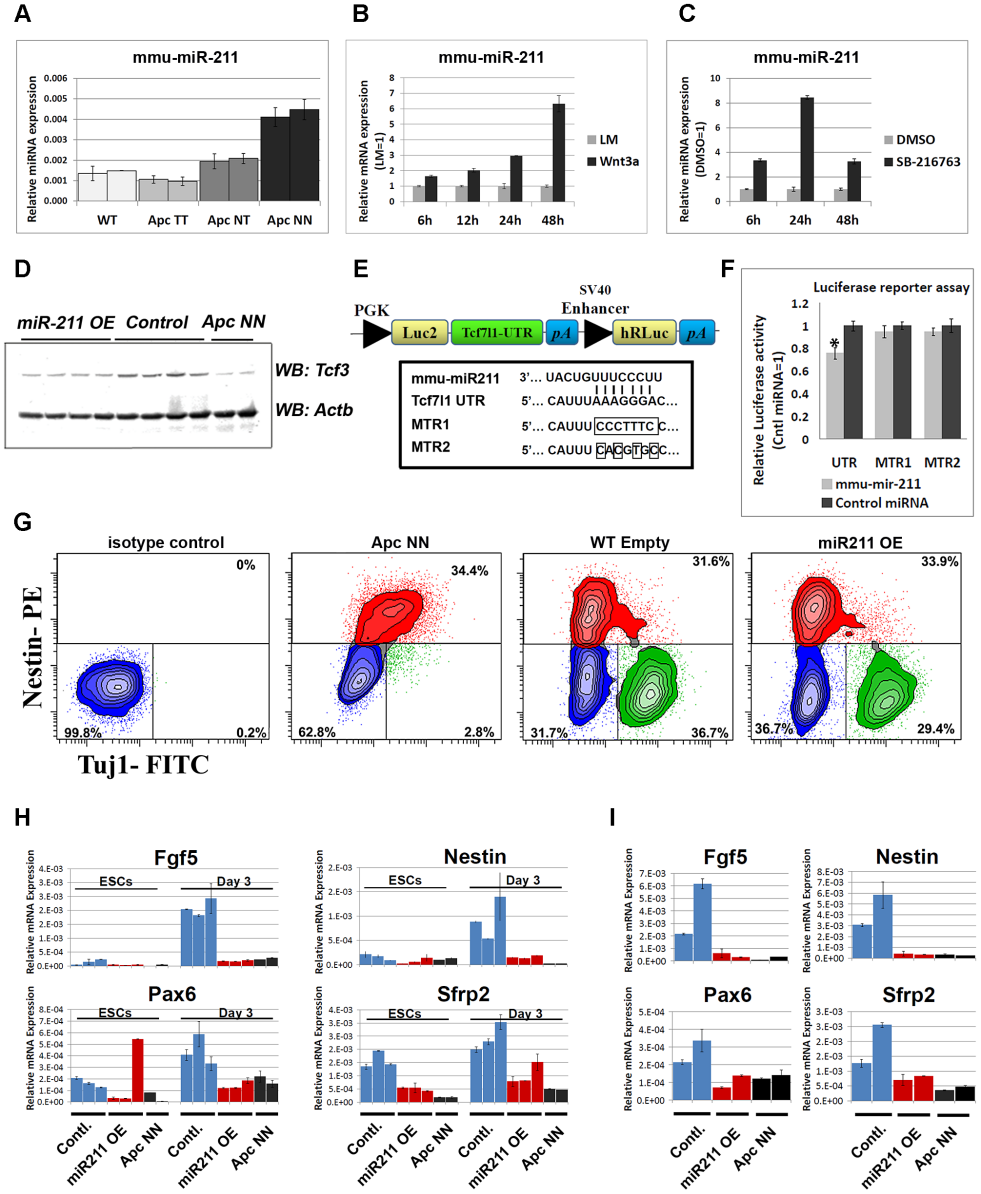
The Wnt-regulated miR-211 targets Tcf3 in mouse ESCs. A. qRT-PCR analysis showing a dosage-dependent up-regulation of miR-211 in different *Apc*-mutant ESCs. SnoRNA-234 was used as an internal control; bars represent n = 2±SD. B–C. Time course analysis of wild type ESCs treated with Wnt3a conditioned medium (B) or with the GSK-inhibitor SB-216763 (C). L-medium and DMSO were used as controls, respectively. RNAs were isolated at different time points and were subjected to qRT-PCR analysis of miR-211 or snoRNA-234 as an internal control. Bars represent n = 2±SD. D. Western blot analysis of Tcf3 expression in protein lysates isolated from independent clones of wild type ESCs stably expressing miR-211 (miR-211 OE) or the corresponding empty vector (control). Two independent *Apc*NN clones were included for comparison. E. Schematic representation of the Tcf3-3′-UTR luciferase vector derived from the pmirGLO construct (Promega). Sequence alignment between miR-211 and its target site on Tcf3-3′-UTR. Site directed mutagenesis was used to introduce 7-bp or 4-bp mutations in Tcf3-3′-UTR. F. HEK-293 cells were co-transfected with the Tcf3-3′-UTR luciferase vector, and either with miR-211 or a non-targeting miRNA. Luciferase activity was measured 24 h post-transfection and normalized to Renilla luciferase signal. The same experiment was repeated with the mutant luciferase vectors, MTR1 and MTR2. Asterix represent *P*-value<0.01 and bars represent n = 3±SEM. G. Flow cytometric analysis of Tuj1 and Nestin in miR-211 over expressing ESCs (miR-211 OE) and their controls (Emp) after 13 days of neural differentiation. Two independent clones were used for each genotype and representative example of each genotype is shown. Numbers in the graph represent the percent of cells in neural (green), progenitor (red) or negative (blue) populations. H. Histogram showing the relative expression of early neural markers *Fgf5*, *Nestin*, *Pax6* and *Sfrp2* in embryoid bodies derived from independent wild type ESCs clones stably expressing miR-211 or the corresponding empty vector. RNAs were isolated at different time points and were analyzed by qRT-PCR for different lineage markers. Bars represent n = 2±SD. I. qRT-PCR analysis of *Fgf5*, *Nestin*, *Pax6* and *Sfrp2* in wild type ESCs stably expressing miR-211 or the corresponding empty vector, cultured for 24 h in N2B27 medium. Bars represent n = 2±SD.

It has been previously shown that members of the core pluripotency circuit are fine-tuned via microRNA-mediated regulation in embryonic stem cells [33], [34], [35], [36], [37]. Therefore we tested the idea whether Wnt-driven repression of *Tcf3* expression might also be mediated, post-transcriptionally, by Wnt-induced miRNAs. To this aim, we profiled the different *Apc*-mutant ESCs for microRNA expression by using a miRNA array encompassing specific probes for all known mouse miRNAs [38] (data not shown). Of the different candidate miRNAs induced upon Wnt activation, mmu-miR-211 showed a Wnt dosage-dependent up-regulation among the different *Apc*-mutant ESCs (Figure 7A). Accordingly, activation of Wnt signaling in wild type ESCs either by Wnt3a conditioned medium (CM) or by GSK3 inhibition, confirmed that miR-211 is a novel Wnt-regulated microRNA in mouse embryonic stem cells (Figure 7B and 7C).

*In silico* analysis with three software packages, namely Miranda [39], Targetscan [40] and PicTar [41], pointed to several potential miR-211 target genes predicted by all three programs. To narrow down the list of potential targets, qRT-PCR analysis was performed on wild type ESCs compared with *Apc*NN (Figure S7A) as well as on wild type ESCs treated with Wnt3a CM (Figure S7B). We excluded those predicted targets that showed up-regulation upon Wnt signaling. Based on these results *Sox11*, *Sf3b1* and *Tcf3* were selected for further analysis. Several stable ESC clones were generated which ectopically over-express miR-211 in an otherwise wild type background (Figure S7C). Western blot analysis showed that, unlike Sox11 and Sf3b1 (Figure S7D), Tcf3 protein level was decreased upon miR-211 ectopic expression (Figure 7D). To confirm that miR-211 directly targets Tcf3, we cloned the 3′ untranslated region (3′UTR) of the mouse *Tcf3* gene in the pmirGLO reporter plasmid (Figure 7E) and performed a luciferase-based reporter assay. Transfection of HEK293 cells with the *Tcf3*-3′UTR reporter plasmid confirmed that *Tcf3* is a direct target of miR-211 (Figure 7F). The inhibitory effects of miR-211 were not observed when mutant forms of the 3′UTR, i.e. lacking 7 or 4 nucleotides of the miRNA seed sequence target (MTR1 and MTR2 respectively) were used (Figure 7F).

We next assessed the differentiation potential of miR-211 over-expressing clones using *in vitro* neural differentiation assay as well as *in vivo* teratoma formation. FACS analysis for Tuj1, a marker for mature neurons and Nestin, revealed that both miR-211 over-expressing ES cells and their wild type controls give rise to similar number of neurons and neural progenitor cells after 13 days of *in vitro* differentiation, thus suggesting that miR-211 does not affect terminal neural differentiation. As expected, *Apc*NN cells show a dramatic reduction in mature, Tuj1-proficient neurons (Figure 7G). Teratoma formation assay also confirmed that miR-211 does not suffice to inhibit neural differentiation (data not shown).

To evaluate the role of miR-211 at earlier stages of differentiation, we derived embryoid bodies (EBs) from miR-211 over-expressing cells and their wild type controls and analyzed lineage differentiation at different time points. EBs derived from wild type ES cells encompass differentiated lineages from the three germ layers, thus providing an *in vitro* assay recapitulating the early steps of embryonic development. qRT-PCR analysis for different lineage-specific markers indicated that, unlike mesodermal, endodermal and pluripotency markers (data not shown), early neuroectodermal differentiation was specifically attenuated by miR-211. We found that expression of the primitive ectoderm marker *Fgf5* and of the neural progenitor markers *Nestin* and *Pax6* as well as the early neural differentiation marker *Sfrp2* were repressed at day 3 of EB formation. Notably, these effects could not be detected at later time points (day 6, 9 or 12; data not shown). Similar results were obtained at early time points (i.e. after 24 h) in N2B27 culture medium, previously described to induce neural differentiation in mESCs [42] (Figure 7H and 7I). These results suggest that miR-211 functions at early stages of neural differentiation and its ectopic expression in wild type ES cells is not sufficient to inhibit further neural commitment as differentiation proceeds.

Altogether, our results indicate that miR-211, a novel Wnt-regulated miRNA, can fine-tune *Tcf3* expression and attenuate early neural differentiation in wild type ESCs.

## Discussion

The role of Wnt/β-catenin signaling in controlling self-renewal and lineage differentiation in pluripotent embryonic stem cells has been a matter of controversy. Although both GSK3 inhibitors and Wnt ligands are essential to support ESCs self-renewal, it is yet unclear whether this occurs through β-catenin- and TCF-dependent mechanisms [43]. Among the members of the Tcf/Lef family of transcription factors, Tcf3 and Tcf1 are the most abundant in ES cells. This is of relevance as, while Tcf1 appears to function as a canonical transcriptional activator upon association with β-catenin, Tcf3 acts as a β-catenin-independent transcriptional repressor of self-renewal, suppressing genes such as *Nanog*, *Oct4* and other members of the core pluripotency circuitry [17], [19]. In this scenario, it is yet unclear how canonical Wnt signaling controls the balance between Tcf1- and Tcf3-mediated gene activation and repression in the regulation of self-renewal and differentiation in ESCs.

During the last few months, several studies have been published on the specific roles of β-catenin and Tcf3 in these processes [4], [5], [6], [44]. In the classical Wnt model, Tcf factors bind DNA and repress gene expression in the absence of active Wnt signaling. Activating the signaling pathway leads to the binding of β-catenin to Tcf proteins thus converting them from transcriptional repressors to transcriptional activators. Among the four members of Tcf/Lef family, Tcf3 seems to be different as its repressor function is not directly affected by Wnt signaling. In this perspective, two modes of action have been described for the relief of Tcf3 repression by Wnt signaling: 1) Tcf3 phosphorylation by homeodomain interacting protein kinase 2 (HIPK2) which is mediated by β-catenin and results in displacement of Tcf3 from its target sites [45]; and 2) direct physical interaction between β-catenin and Tcf3 which displaces Tcf3 and inhibits its repressive role in the context of active Wnt signaling [6], [46]. Recently, using a knock-in mouse model lacking the β-catenin-interaction domain of Tcf3, Wu et al have demonstrated that counteracting Tcf3 function is not mediated by the physical interaction between β-catenin and Tcf3 during the first stages of embryonic development [47]. In view of these models, our data suggest that transcriptional and post-transcriptional down-regulation of Tcf3 expression might be yet another mechanism by which Wnt signaling inhibits Tcf3 function. It is worthwhile mentioning, however, that Wnt signaling does not seem to fully suppress Tcf3 expression and that residual levels of Tcf3 are retained even in the most severely truncated *Apc* mutant alleles (i.e. *Apc* ^Min/Min^ ESCs; Figure 2A) which encode for extremely high Wnt signaling dosages. Altogether these observations suggest that Wnt/β-catenin signaling regulates Tcf3 at several levels and by a combination of multiple mechanisms during different stages of embryonic development.

Although over-expression of a dominant negative form of Tcf1 or Tcf4 reduced the canonical Wnt reporter activity (TOP-Flash), it failed to rescue the neural differentiation in GSK-null ESCs [25]. Inhibition of β-catenin in GSK3β-null ESCs, however, was sufficient to rescue the neural differentiation defect thus confirming the central role of β-catenin-dependent mechanisms in this process [25]. The partial rescue of neural differentiation by *Tcf3* expression in *Apc*NN cells, as shown here, highlights the distinct role of Tcf3 from other members of the Tcf/Lef family and suggests that a plethora of Tcf3-dependent and -independent mechanisms underlie the Wnt-regulated lineage differentiation in embryonic stem cells.

As for self-renewal maintenance in ES cells, Wray et al have shown that a mutant form of β-catenin where the trans-activating domain was deleted, can still maintain self-renewal in mESCs cultured in 2i medium [6]. This suggests that maintenance of self-renewal is mediated by Tcf3 displacement rather than β-catenin signaling in 2i culture. Based on this, one can hypothesize that forced overexpression of *Tcf3* in Wnt context could restore the dependency on CHIRON in serum-free culture. Our data show that *Tcf3* overexpression in *Apc*NN cells does not induce differentiation in 2i culture, highlighting the dominant role of Wnt signaling in this process. This is in line with the report by Yi et al. which showed that over expressing Tcf3 in the context of Wnt signaling activation has minimal effect on self-renewal suggestive of a synergistic action of Tcf3 antagonism and β-catenin/Tcf1 signaling [5].

In an attempt to elucidate the mechanisms underlying Tcf3 downregulation in the context of active Wnt signaling, we found that Tcf3 down-regulation does not require DNA methylation but is associated with alterations in histone marks at the core Tcf3 promoter region which are likely to regulate Tcf3 expression. Notably, these modifications occur shortly after Wnt stimulation and it is plausible to think that the chromatin modifications within the *Tcf3* locus can trigger the downregulation process of *Tcf3* expression which can be stabilized further on via miR-211 function. Epigenetic regulation through histone modification or DNA methylation was also shown previously for other antagonists of Wnt signaling such as *DACT3*, *sFRPs*, *WIF1 and DKK-1* in different cancer cells [48], [49], [50], [51]. Further experiments are required to clarify whether this mode of gene repression is a general mechanism for Wnt-induced gene silencing in embryonic stem cells and tumor cells. Although the mediator of the observed chromatin modifications downstream of Wnt signaling remains elusive, we found that the putative *cis*-acting element, if any, is not located in the 6.7 kb promoter region which was previously described to regulate Tcf3 expression in different cell types [31]. Further work is needed to identify and study these *cis*-acting elements which might be of potential interest for providing further insight into the transcriptional repression downstream of Wnt signaling.

As an additional regulatory mechanism, we also described a novel Wnt-induced micro RNA, miR-211, and demonstrated that it targets Tcf3 in *Apc*NN ESCs. However, miR-211 over-expression in wild type ESCs does not reduce Tcf3 levels to the same degree as observed in *Apc*NN ES cells thus suggesting that multiple Wnt-mediated mechanisms are likely to exist. On the other hand, microRNAs usually exert their function by targeting multiple genes and it is plausible that miR-211 inhibits early neural differentiation in mESCs by repressing target genes other than *Tcf3*. Further experiments are required to characterize the loss of miR-211 function phenotype in mouse ESCs in order to evaluate the long-term effects on neural differentiation. The observation that Wnt signaling induces miR-211 expression might also be of interest for other disciplines of research and in particular cancer. In line with our observation, a tumor promoting function has recently been described for miR-211 in colorectal cancer cells [52]. Accordingly, miR-211 has also been shown to play a key role in melanoma tumor formation and metastasis, as well as mesenchymal to epithelial transition (MET) [53], [54], [55]

Taken together, we have revealed two downstream effects of Wnt signaling which contribute to the differentiation defects observed upon constitutive activation of canonical Wnt signaling, namely downregulation of Tcf3 expression and induction of miR-211. These cooperatively contribute to the inhibition of neural differentiation previously observed in *Apc*-mutant mouse ESCs [1]. We suggest that Wnt signaling represses *Tcf3* expression possibly by altering the histone marks at the Tcf3 promoter and by activating miR-211 expression, thus extending our understanding of Tcf3 regulation in stem cells. In the future, additional studies are required to elucidate how these mechanisms contribute to the regulation of *Tcf3* expression and, more in general, how Wnt signaling regulates stemness in embryonic and adult stem cells.

## Materials and Methods

### Ethics statement

This study was carried out in strict accordance with the recommendations in the Guide for the Care and Use of Laboratory Animals of the National Institutes of Health. The protocol was approved by the Committee on the Ethics of Animal Experiments of the Erasmus Medical Center (DEC permit numbers EMC 2351). All efforts were made to minimize suffering.

### ES cell cultures and expression vectors

*Apc* ^1638N/+^ and *Apc* ^1638T/+^ animals, kept on an inbred C57Bl6/J background, were bred to derive ES cells from pre-implantation blastocyts according to previously described protocols [56]. Cells were cultured on MEFs inactivated by Mitomycin-C (Sigma) in Dulbecco's Modified Eagle's Medium (DMEM, Gibco) supplemented with 10% fetal calf serum (FCS, Gibco), L-glutamine (2 nM, Gibco), Na-Pyruvate (1 mM, Gibco), non essential amino acids (0.1 mM each, Gibco), 2-mercaptoethanol (55 µM, Gibco) and LIF (1000 U/ml, Milipore). Bruce 4 ESCs were purchased from American Type Culture Collection (ATCC) and *Tcf3^−/−^* and their wild type control GS1 ESCs were obtained as previously decribed [14]. To stimulate Wnt signaling in wild type ESCs, cells were cultured on gelatin coated dishes and treated with Wnt3a-conditioned medium (collected from L-cells expressing Wnt3a plasmid) or L-control medium (collected from parental L-cells). Conditioned media were diluted 1∶1 with ES medium and added to wild type ESCs for different time points. The Gsk-inhibitor SB-216763 was purchased from Sigma, dissolved in DMSO and used at 10 µM final concentration. DMSO was used as control in all the experiments.

Stable clones over expressing mmu-miR-211 were generated by transfecting Bruce4 wild type ESCs with miR-211 expressing plasmid pEZX-MR01 (Genecopoeia), or the corresponding empty vector. Several G418 resistant clones (200 µg/ml) were selected and validated for miRNA expression. In order to generate Tcf3 over expressing ESCs, *Apc*NN ESCs were co-transfected with pCAG-HA-*Tcf3*-IRES-EGFP (gift of Dr. Bing Lim, National University of Singapore, Singapore,) and Hygromycin resistance plasmid. Transfected ES cells were selected for Hygromycin (150 µg/ml). GFP expression in resistant clones was employed for validation purposes. Several independent clones were isolated and, upon validation by qPCR and western blot analysis for Tcf3 expression, employed for subsequent experiments.

The *Tcf3*-3′-UTR plasmid was obtained by PCR amplification from mouse genomic DNA of a 565 bp fragment encompassing the *Tcf3*-3′-UTR inclusive of the miR-211 target site (forward primer 5′-AAATTGAGCTCTCCCCTTGCGCTGTGGTG-3′; reverse primer 5′-AAAAACTCGAGGGTGGGGGAAGGGGCAGA-3′). PCR products were digested with *Sac*I and *Xho*I and ligated into *Sac*I and *Xho*I-cut pmirGlo plasmid (Promega). All constructs were sequenced to verify their authenticity.

### Microarray analysis

RNA was isolated using the RNeasy Mini Kit (QIAGEN) from cells lysed directly on the plate; a DNase step on the column was performed according to manufacturer's instructions. RNA quality was controlled by RNA 6000 Nano LabChip kit (Agilent Technologies). RNA was labeled using the GeneChip One-Cycle Target Labeling kit, hybridized to MOE430 2.0 arrays (Affymetrix) according to manufacturer's instructions. For data analysis, CEL files were uploaded and normalized using MAS 5.0 algorithm in Expression Console software (Affymetrix, Inc). Expression analysis was performed using Partek Genomics Suite 6.5 ((Partek Inc., St. Louis, MO) and Excel 2010 (Microsoft). A robust empirical method coupled with a validation step using qRT-PCR was used to confirm the modulation of gene expressions between different genotypes. A modulation of gene expression was validated when the observed fold-change is ≥1.5 and corresponding to none overlapping individual values, not present in the background. The unsupervised hierarchical clustering was performed after MAS 5.0 normalization, using Pearson's dissimilarity as distance measure and Ward's method for linkage analysis.

### DNA methylation analysis

ESCs were cultured on 0.1% gelatin-coated dishes without MEFs for 2 passages and genomic DNA was isolated using DNeasy Blood & Tissue Kit (Qiagen). 1 µg of genomic DNA was used in bisulfite conversion reaction using EZ DNA Methylation Kit (Zymo Research) according to the manufacturer's instructions. Converted DNA was amplified by PCR using specific primers (Table S2) designed with Methyl Primer Express Software 1.0 (Applied Biosystems) or MethPrimer software [57].

The PCR amplification was carried out using KAPA2G Robust HotStart Taq DNA polymerase (Kapa biosystems) and PCR conditions were: 95°C for 3 min and 39 cycles of 95°C for 15 s, 57 or 53°C for 15 sec and 72°C for 15 sec, followed by 10 min at 72°C. PCR products from Region A, B and D were employed in direct sequencing using ABI BigDye Terminator and ABI 3130×l genetic analyzer (Applied Biosystems).

### Embryoid body formation

ESCs were trypsinized, re-suspended in ES medium and plated on gelatin-coated culture dishes for 30 min to remove MEFs. Non-attached ESCs were resuspended in EB medium (ESCs medium without LIF) at a cell density of 2×10^5^ cells/ml and plated on a non-adherent bacterial dish to initiate EB formation. EBs were collected by centrifugation (800 rpm for 5 min) every three days and re-suspended in fresh medium. 1/5 volume of EBs suspension was used for RNA extraction while the remaining EBs were kept in culture until day 12.

### N2B27 short term differentiation

Differentiation assays were performed as previously described [42]. Shortly, cells were trypsinized and plated on gelatine-coated dishes in N2B27 medium consisting of DMEM/F12∶Neurobasal medium (1∶1, Gibco) supplemented with N2 and B27 (Gibco). Cells were harvested after 24 h or 48 h of differentiation for further analysis.

### −4/+4 neural differentiation assay

Neuronal differentiation of ESCs was induced as previously described [30]. Briefly, ESCs were trypsinized and incubated in ES medium on gelatine-coated dishes for 30 min. to allow attachment of MEFs. Non attached cells were collected and 3×10^6^ cells were cultured in 10 cm. non-adherent bacterial dishes (Greiner Bio-One) in EB medium for 8 days. Medium was refreshed every 2 days and 5 µM all-trans retinoic acid (Sigma) was added at day 4 and 6. On day 8 cells were trypsinized and plated on poly-L-ornithine/laminin-coated dishes at a density of 2×10^5^ cells/cm^2^ in N2 medium. Poly-L-ornithine (Sigma) and laminin (Roche) were used at final concentrations of 0.1 mg/ml and 20 µg/ml, respectively.

N2 medium was refreshed after 2 and 24 hrs. from cell plating to remove dead cells. The N2 medium consisted of: DMEM/F12 (Gibco) supplemented with L-glutamine (Gibco), Nonessential amino acids (GIBCO), Insulin (25 ug/ml, Sigma), Progesterone (20 nM, Sigma), Putrescine (100 nM, Sigma), Transferrin (50 µg/ml, Sigma), Bovine serum albumin (50 µg/ml, Sigma), Sodium selenite (30 nM, Sigma) and Penicillin-Sterptomycin (Gibco).

After 48 h from cell plating, medium was changed to N2B27 and refreshed every 2 days. Cells were collected after 5 days of plating for further analysis.

### Colony forming assay and alkaline phosphatase staining

Cells were trypsinized and plated on 0.1% gelatin-coated dishes for 30 min to remove MEFs. 500 FACS sorted cells were plated on each well of a gelatinized 24-well plate in N2B27 medium supplemented with different combinations of CHIR99021 (3 µM, Stemgent), PD0325901 (1 µM, Stemgent) and LIF (1000 U/ml, Milipore). Total number of colonies were counted after 5 days from plating upon staining with alkaline phosphatise (Milipore) according to manufacture's instructions.

### Teratoma formation

Teratomas were obtained upon subcutaneous injection of 5×10^6^ cells (in PBS) into C57Bl6/J, for *Apc*-mutant ESCs (and their wild type controls), and NOD/SCID, for *Tcf3* ^−/−^ ESCs (and their wild type controls), recipient mice. Teratomas were collected after 2–3 weeks and used for further experiments.

### RNA isolation, cDNA synthesis, and qRT–PCR

RNA was isolated using Trizol (Invitrogen) or RNeasy Mini Kit (QIAGEN) and treated with DNase (Ambion) to remove contaminating genomic DNA. For gene expression analysis cDNA was synthesized using 1 µg RNA and the RevertAid H Minus First Strand cDNA Synthesis Kit (Thermo). microRNA expression analysis was performed using 40 ng of total RNA isolated by Trizol and employed in cDNA synthesis reaction using TaqMan MicroRNA Reverse Transcription kit (ABI). Real-time RT-PCR was performed using Applied Biosystems inventoried assays or TaqMan MicroRNA Assays on a 7900HT ABI real-time PCR system (Applied Biosystems). The Delta-Ct method was used to quantify the mRNA or miRNA relative gene expressions. *Actb* or *snoRNA234* were used for normalization, respectively. qPCR analysis of the selected genes were performed using Fast SYBR Green Master Mix (ABI) and the primers listed in Table S2.

### Immunohistochemistry

Isolated teratomas were fixed in PFA (4%) and embedded in paraffin. Five µm sections were mounted on slides stained by H&E for routine histology. Antibodies employed for IHC analysis included: rabbit anti-GFAP (1∶5000, Z0334, DAKO,); mouse 2H3 against Neurofilaments (1∶50, Developmental Studies Hybridoma Bank); mouse SV2 against Synaptic vesicles (1∶50, Developmental Studies Hybridoma Bank); mouse A4.1025 against Adult myosin (1∶50, Developmental Studies Hybridoma Bank); goat anti-Oct3/4 (1∶100, sc-8629, Santa Cruz). Signal detection was performed using HRP-conjugated Goat anti mouse (1∶250, Jackson ImmunoResearch), rabbit-anti-Goat-HRP (Dako) or Rabbit Envision kit (Dako).

### Immunofluorescence and confocal microscopy

Cells were harvested and fixed in 2% PFA for 20 min, washed with PBS, and permeabilized with 0.1% triton X-100 in PBS for 15 minutes. Cells were then incubated in Blocking solution (PBS, 4% FCS) for 30 min., stained overnight at 4°C with the primary antibody, washed and finally incubated with the secondary antibody for 2 h. Confocal analysis was performed with a Zeiss LSM510 confocal microscope. Tuj-1-Alexa488 was detected using a 488 nm laser and BP 500–550 emission filter. DRAQ5 was detected using a 633 nm laser and LP650 nm emission filter. Alexa 488-conjugated monoclonal anti-Tuj-1 was from Covance (A488-435L) and was used at 1∶4000 dilution.

### Flow cytometry analysis

Flow cytometric analysis was performed with a BD FACSAria III, using a yellow-green laser at 561 nm and a BP582/15 emission filter to detect anti-Nestin-PE antibodies, and 488 nm laser and LP502 and BP530/30 emission filters for Tuj-Alexa-488 antibodies. A Live-Dead-Fixable red staining (Invitrogen) was performed before fixation, to exclude dead cells and was detected using a 633 nm laser and BP660/20 emission filter.

Alexa 488-conjugated anti-Tuj-1 antibody was used at a 1∶4000 dilution and the mouse anti-nestin antibody was from BD (556309) and was used at a 1∶500 dilution together with a 2^nd^ Rat-anti-mouse PE-conjugated antibody (BD; 1∶1000). DRAQ5 was from Biostatus and was used as recommended by the manufacturer.

### Luciferase reporter assays

For the β-catenin/TCF reporter assay, 5×10^5^ ES cells were plated on 24-well plates seeded with MEFs and subsequently transfected by Fugene HD (Roche) with 250 ng of the TOP-Flash or FOP-Flash reporter constructs [28] together with 25 ng of the Renilla luciferase vector for normalization purposes. Luciferase activity was measured by Dual–Luciferase Reporter Assay System (Promega). Tcf3 promoter activity was evaluated in *Apc*NN and wild type ESCs similar to β-catenin/TCF reporter assay, as mentioned above and by using Tcf3-promoter constructs (kindly provided by Nina Solberg, SCI-CAST Innovation Center, Norway) and pGL3 empty vector a control. To examine the effect of Wnt3a treatment on Tcf3 promoter activity, cells were transfected with luciferase constructs and treated with Wnt3a condition medium or L-control medium for 48 h and luciferase activity was measured using Dual–Luciferase Reporter Assay System (Promega).

For the 3′UTR-Luciferase reporter assay, HEK293 cells were plated in 24-well plates at a density of 0.5×10^5^ cells per well. Cells were co-transfected with 250 ng of UTR (or MTR) reporter plasmid and either mmu-miR-211 mimic or non-targetting oligos (40 nM, Dharmacon) using lipofectamin 2000 (Invitrogen). Twenty-four hrs. after transfection, firefly-luciferase activity was measured by Dual–Luciferase Reporter Assay System (Promega) and normalized to the co-expressed Renilla luciferase signal.

### Western blot analysis

ES cells were lysed using Cell Lysis Buffer (9803, Cell Signaling) and a cocktail of protease inhibitors (11836170001, Roche). Subsequently, NuPage LDS Sample Buffer (NP0008, Invitrogen) and DTT (1 mM) were added. Primary antibodies employed in western blot analysis included: Tcf3 (sc-8635, Santa Cruz); Sox2 (AF2018, R&D Systems); Sox11 (sc-20096, Santa Cruz); Oct4 (sc-5279, Santa Cruz); Dyrk1A G-19 (G2905, Santa Cruz); Sap155/Sf3b1 (D138-3, MBL); Nanog (AB5731, Milipore); β-actin (A5441, Sigma); β-tubulin (ab6046, Abcam). Lysates were loaded on 10% SDS-PAGE (BIO-RAD System), and transferred onto Immobilon-FL PVDF membrane (IPFL00010, Millipore). Blocking was performed at room temperature using LI-COR Blocking buffer (Part#927-40000) diluted 1∶1 with PBS. Incubation with the first antibody was performed overnight at 4°C. Blots were subsequently incubated with fluorescent-labeled secondary antibodies for 30 min. at room temperature. Goat anti-mouse IgG – IRDye 680 (1∶5000, LI-COR Biosiences), Goat anti-rabbit IgG – IRDye 800CW (1∶5000, LI-COR Biosiences) and Donkey anti-goat-IRDye 800CW (1∶5000, LI-COR Biosiences) were used as secondary antibodies. Fluorescent signal was detected using LI-COR scanner (LI-COR Biosiences).

### Site-directed mutagenesis

Two mutant forms of the Tcf3-3′UTR-luc plasmid were generated using QuikChange Lightning Site-Directed Mutagenesis Kit (Agilent, 210518). We introduced either 7 bp substitutions in the miRNA binding site (AAAGGGA into CCCTTTC) to generate the MTR1-Luc plasmid, or 4 bp (AAAGGGA into cAcGtGc) to generate the MTR2-Luc plasmid. The following mutagenesis primers were employed in the reaction:

For MTR1, sense primer is 5′-tctgaaatggtccccccccctgcatttccctttcctcaaggtgcctaccactgccttc-3′ and antisense primer is 5′-gaaggcagtggtaggcaccttgaggaaagggaaatgcagggggggggaccatttcaga-3′. For MTR2 plasmid the sense primer is 5′-gtccccccccctgcatttcacgtgcctcaaggtgcctacc-3′ and the antisense primer is 5′-ggtaggcaccttgaggcacgtgaaatgcagggggggggac-3′.

The mutagenesis reaction was performed according to manufacture's instruction. Briefly, mutant strands were synthesized using the described primers followed by DpnI digestion of the amplification products to remove the parental methylated strands. Digestion reactions were transformed in XL10-Gold Ultracompetent cells and bacterial clones with correct nucleotide substitutions were used for further plasmid extraction.

### Chromatin immunoprecipitation (ChIP)

ChIP was performed on wild type and *Apc*NN ESCs or on wild type ESCs treated for 12 h with Wnt3a conditioned medium or L-control medium (1∶1 dilluted with ES medium). Briefly, cells were fixed in 1% PFA for 30 minutes at room temperature and PFA was quenched afterwards with 125 mM glycine. Cells were washed with buffer B (0.25% Triton-X 100, 1 mM EDTA, 0.5 mM EGTA, 20 mM Hepes, pH 7.6), buffer C (150 mM NaCl, 1 mM EDTA, 0.5 mM EGTA, 20 mM Hepes, pH 7.6). Cells were then sonicated in ChIP incubation buffer (0.3% SDS, 1% Triton-X 100, 0.15 M NaCl, 1 mM EDTA, 0.5 mM EGTA, 20 mM Hepes, pH 7.6) using a BioRuptor sonicator (Cosmo Bio Co., Ltd) to obtain DNA fragments 200–700 base pairs. Chromatin was diluted in ChIP dilution buffer (with 0.15% SDS) and incubated with BSA-blocked protein-A/G Sepharose beads (Amersham) and 5 µg antibody overnight at 4°C. Antibodies used in this study include: H3K4me3 (Abcam, Ab8580-50), H3K27me3 (Upstate, 07-449), H3K9me3 (Abcam, Ab8898-100), H3Ac (Millipore #06-599)

Beads were washed with buffer 1 (0.1% SDS, 0.1% deoxycholate, 1% Triton-X 100, 150 mM NaCl, 1 mM EDTA, 0.5 mM EGTA, 20 mM Hepes pH 7.6), buffer 2 (0.1% SDS, 0.1% deoxycholate, 1% Triton-X 100, 0.5 M NaCl, 1 mM EDTA, 0.5 mM EGTA, 20 mM Hepes pH 7.6), buffer 3 (250 mM LiCl, 0.5% deoxycholate, 0.5% NP-40, 1 mM EDTA, 0.5 mM EGTA, 20 mM Hepes, pH 7.6), and buffer 4 (1 mM EDTA, 0.5 mM EGTA, 20 mM Hepes, pH 7.6). Chromatin was eluted for 30 min at room temperature in elution buffer (1% SDS, 0.1 M NaHCO_3_) and together with input chromatin, decrosslinked overnight at 65°C in the presence of 200 mM NaCl. DNA was extracted using QIAquick PCR Purification Kit and was used in QPCR analysis using Fast SYBR Green Master Mix (ABI) and primers indicated in Table S2.

## Supporting Information

Figure S1 qRT-PCR validation of microarray results. Selected differentially expressed genes include Wnt and pluripotency-related genes. Measurements were performed in duplicates and using two independent cell lines per genotype. *Actb* was used for normalization. Plots represent average ± SD of normalized qRT-PCR values for two independent clones of each genotype.
(TIF)

Figure S2 A. Histogram showing relative expression of *Axin2* and of members of the Tcf/Lef family in wild type, *Apc*NN and *Apc* ^Min/Min^ ESCs. *Actb* was used for normalization. Bars represent n = 2±SD. B–C. qRT-PCR analysis of *Axin2* and of members of the Tcf/Lef family in wild type ESCs treated for different time intervals with Wnt3a conditioned medium (B) and with the GSK inhibitor SB-216763 (C). L-medium and DMSO were used as control media. *Actb* was used for normalization. Bars represent n = 2±SD.
(TIF)

Figure S3 Heat map showing the results of the qRT-PCR validation of microarray data relative to selected genes. Genes differentially expressed between *Apc*NN and wild type ESCs were compared to the list of genes differentially expressed between ApcNN and Tcf3OE cells (Table S3). Among several genes overlapping between the two microarray studies, 15 were selected for QPCR validation. The heat map shows the fold change values obtained from the microarray (M) and qRT-PCR (Q) data. *Apc*NN/WT values represent the average fold change of 2 *Apc*NN versus 2 WT ES clones for each gene. Tcf3 OE/*Apc*NN values represent the average fold change of three Tcf3 OE versus three *Apc*NN clones (parental cells as well as empty vector transfected cells) for each gene. Scale represents log_2_ values.
(TIF)

Figure S4 Supporting data to Figure 3. Flow cytometric analysis of wild type (WT) ESCs cells stained with isotype controls or Nestin and Tuj1 specific antibodies. The double staining allows the identification of Nestin positive neural progenitors and Nestin-negative/Tuj-positive mature neurons (highlighted in green). The left panel shows the Nestin-PE versus Tuj1 and the right panel indicate the Nestin-PE against forward scattering (FSC) of the same sample. Since Tcf3 over expressing clones gave rise to 0.1% mature neurons in average, the Nestin-PE versus FSC has been used in Figure 3.
(TIF)

Figure S5 A. Tcf3 promoter activity in Wnt high and Wnt low ESCs. Luciferase constructs containing different Tcf3 promoter fragments were co-transfected with Renilla luciferase and the relative promoter activity is shown after normalization to Renilla-luciferase values. To monitor the effect of Wnt signaling on Tcf3 promoter activity, luciferase constructs were transfected in wild type ESCs, followed by 24 h treatment with Wnt3a-condition medium (Wnt3a) or L-control medium (LM). Similarly, luciferase constructs were transfected in 2 independent clones of *Apc*NN or wild type ESCs and promoter activity was measured after 48 h of transfection. The genomic location of different Tcf3 promoter fragments is depicted in the scheme. Bars represent n = 2±SD. B. DNA methylation analysis of Tcf3 promoter. Schematic representation of the mouse *Tcf3* promoter defined by the 5′UTR and ∼2 kb large CpG island extending into exons 1–3. For the purpose of methylation analysis, the CpG island was subdivided into regions A, B, C and D. Genomic DNA was first bisulfite-converted and the individual regions either employed in bisulfite-specific PCR followed by DNA sequencing (region A, B and D), or used in methylation-specific PCR assays (region C). Arrows represent methylated (and un-methylated) specific primers for region C. PCR products from regions A, B and D were obtained from *Apc*NN, wild type and *Apc*N/+ (*Apc^1638N/+^*) ESCs and directly sequenced. *Apc*N/+ ESCs were employed as controls since they express similar *Tcf3* levels compared to wild type ESCs (data not shown). The sequencing results are depicted as open and solid circles for unmethylated and methylated CpG dinucleotides, respectively. Dashed circles represent CpGs which were not included in the PCR products. Because of its extremely GC-rich sequence, PCR amplification of region C was carried out using methylated- and unmethylated-specific primers (MSP and USP) covering 4 different CpG dinucleotides. Control DNA (mouse genomic DNA where all CpGs sites are enzymatically methylated by means of CpG Methylase) was used in all bisulfite reactions as a positive control.
(TIF)

Figure S6 Transient activation of Wnt signaling in wild type ESCs reduces H3Ac and H3K4me3 activating marks in Tcf3 promoter. Bruce 4 wild type ESCs were cultured on gelatin-coated dishes and treated with Wnt3a condition medium or L-control medium for 12 h. Cells were used for ChIP-QPCR as described before. Values from each amplicon were normalized to input chromatin. Since no amplification was detected at the negative region (P1) from some of the immunoprecipitated chromatin, values are shown as percent of input DNA. Bars represent n = 2±SD.
(TIF)

Figure S7 A. Histogram showing relative expression of selected miR-211 predicted targets in *Apc*NN and wild type ESCs. Two independent clones were used for each genotype. *Actb* was used for normalization. Bars represent n = 2±SD. B. Histogram showing relative expression of selected miR-211 predicted targets in wild type ESCs treated with Wnt3a condition medium or L-medium for different time intervals. The ratios of Wnt3a CM/L-medium are shown in the graphs. Bars represent n = 2±SD. C. qRT-PCR analysis of miR-211 expression in wild type ESCs stably expressing miR-211 or the corresponding empty vector. Two independent *Apc*NN ESC clones were included for comparison. *snoRNA-234* was used for normalization.Bars represent n = 2±SD. D. Western blot analysis of the miR-211 predicted targets Sox11 and Sf3b1 in miR-211 over expressing cells and their wild type controls.
(TIF)

Table S1 Differentially expressed genes between WT, *Apc*TT, *Apc*NT and *Apc*NN ESCs.
(ZIP)

Table S2 Primer sequences used in QPCR and DNA methylation analysis
(XLS)

Table S3 Differentially expressed genes between *Apc*NN and *Apc*NN-over expressing Tcf3 (Tcf3 OE) ESCs.
(XLS)
