# Supplementary figures and images for: Wnt Signaling Regulates the Lineage Differentiation Potential of Mouse Embryonic Stem Cells through Tcf3 Down-Regulation
Source: PLoS Genet. 2013 May 2;9(5):e1003424. doi: 10.1371/journal.pgen.1003424 (PMC3642041; doi:10.1371/journal.pgen.1003424)

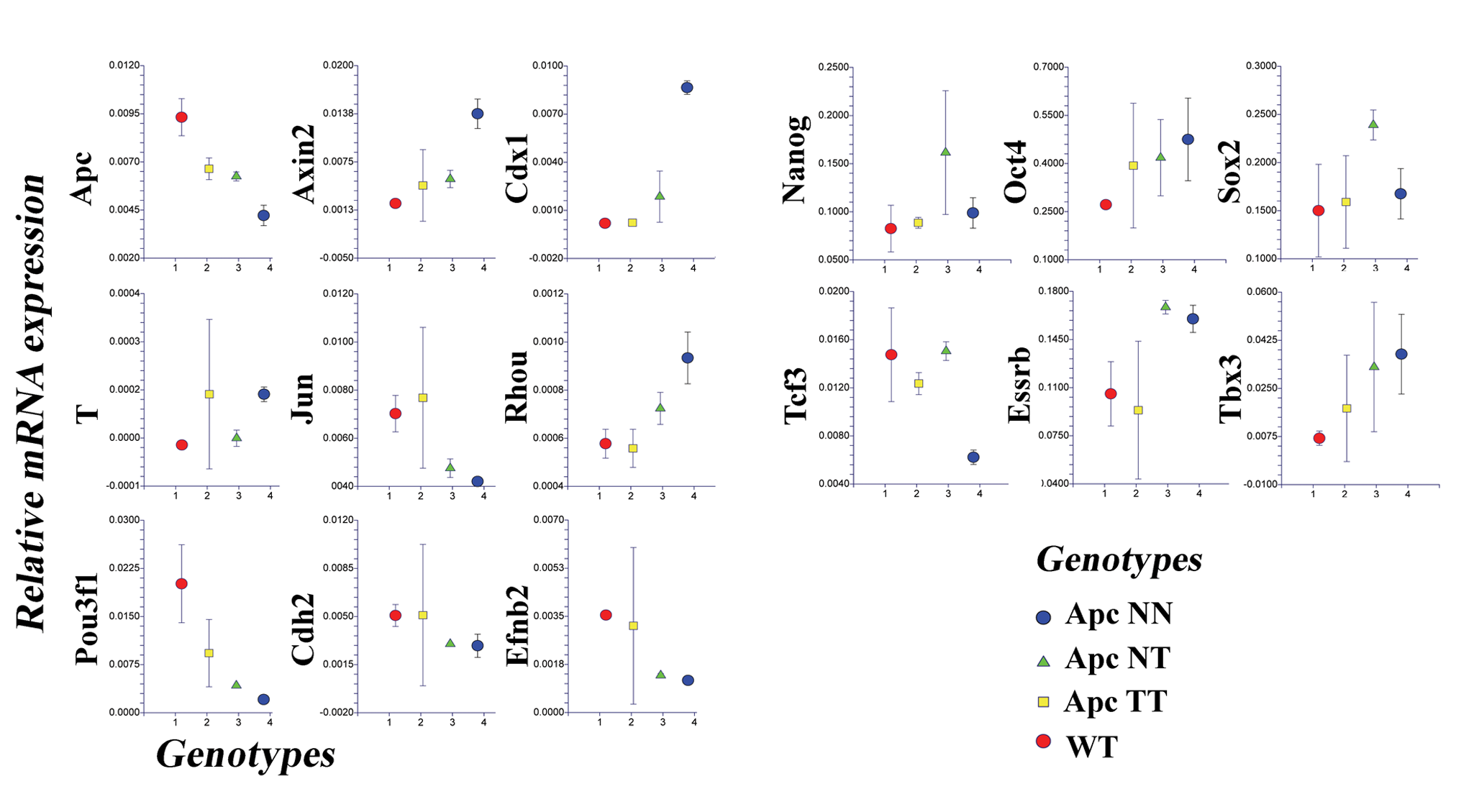

Supplement: Figure S1 — qRT-PCR validation of microarray results. Selected differentially expressed genes include Wnt and pluripotency-related genes. Measurements were performed in duplicates and using two independent cell lines per genotype. Actb was used for normalization. Plots represent average ± SD of normalized qRT-PCR values for two independent clones of each genotype. (TIF) [file pgen.1003424.s001.tif]

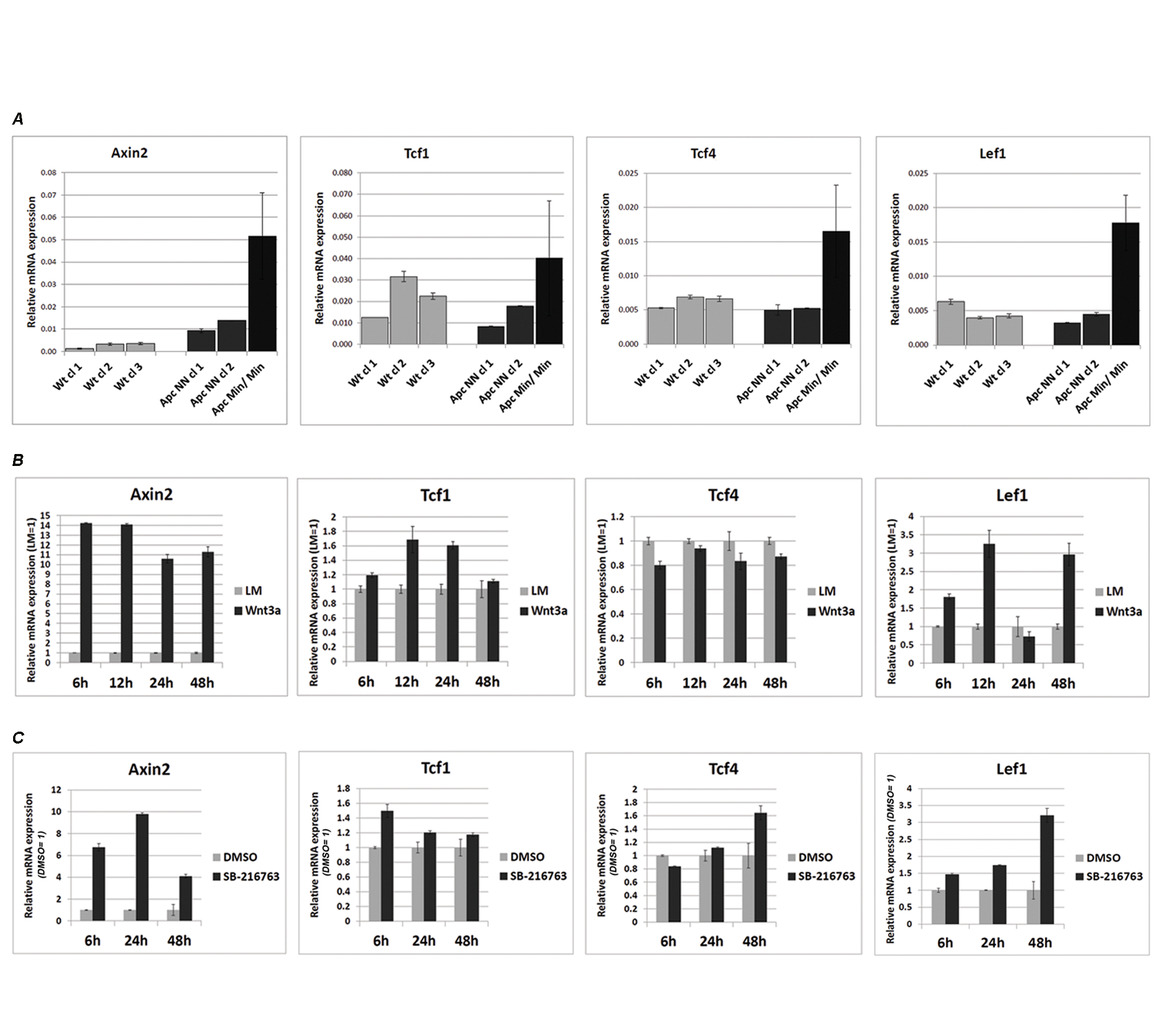

Supplement: Figure S2 — A. Histogram showing relative expression of Axin2 and of members of the Tcf/Lef family in wild type, ApcNN and Apc Min/Min ESCs. Actb was used for normalization. Bars represent n = 2±SD. B–C. qRT-PCR analysis of Axin2 and of members of the Tcf/Lef family in wild type ESCs treated for different time intervals with Wnt3a conditioned medium (B) and with the GSK inhibitor SB-216763 (C). L-medium and DMSO were used as control media. Actb was used for normalization. Bars represent n = 2±SD. (TIF) [file pgen.1003424.s002.tif]

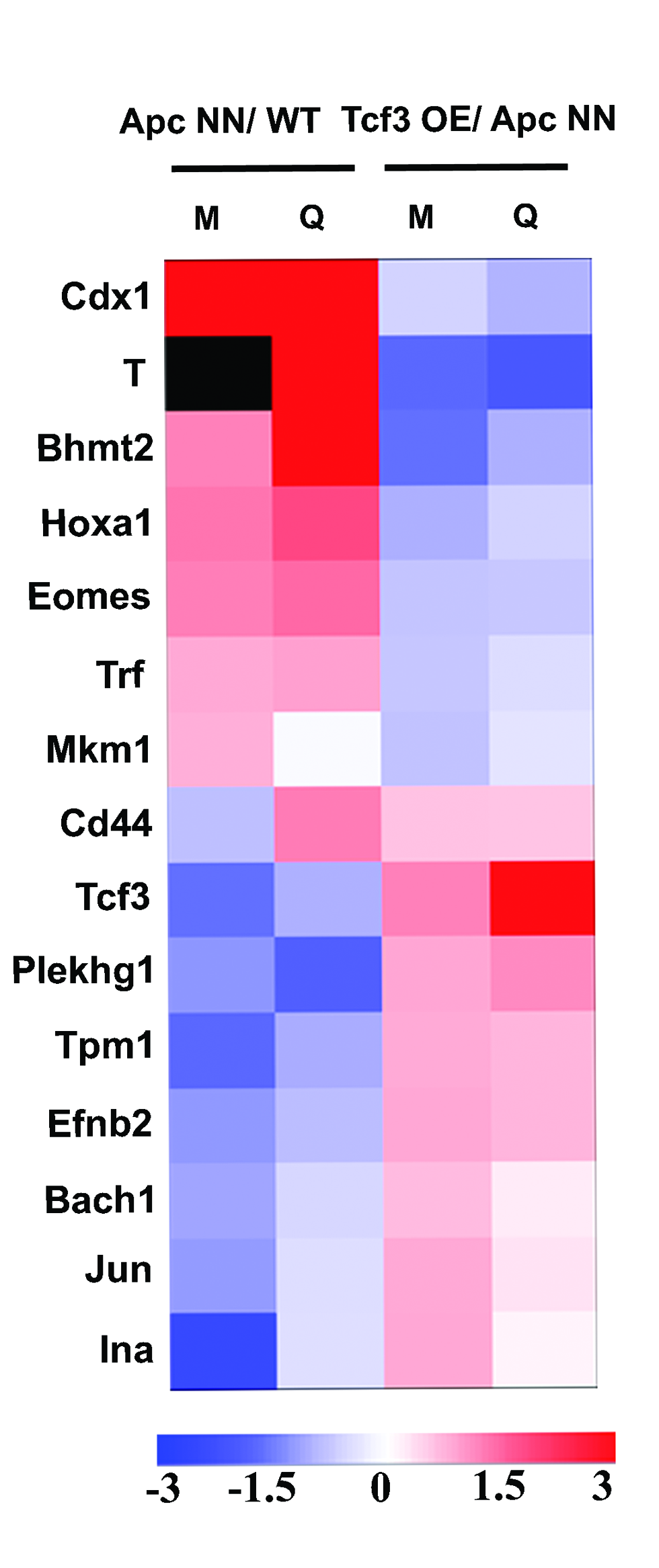

Supplement: Figure S3 — Heat map showing the results of the qRT-PCR validation of microarray data relative to selected genes. Genes differentially expressed between ApcNN and wild type ESCs were compared to the list of genes differentially expressed between ApcNN and Tcf3OE cells (Table S3). Among several genes overlapping between the two microarray studies, 15 were selected for QPCR validation. The heat map shows the fold change values obtained from the microarray (M) and qRT-PCR (Q) data. ApcNN/WT values represent the average fold change of 2 ApcNN versus 2 WT ES clones for each gene. Tcf3 OE/ApcNN values represent the average fold change of three Tcf3 OE versus three ApcNN clones (parental cells as well as empty vector transfected cells) for each gene. Scale represents log2 values. (TIF) [file pgen.1003424.s003.tif]

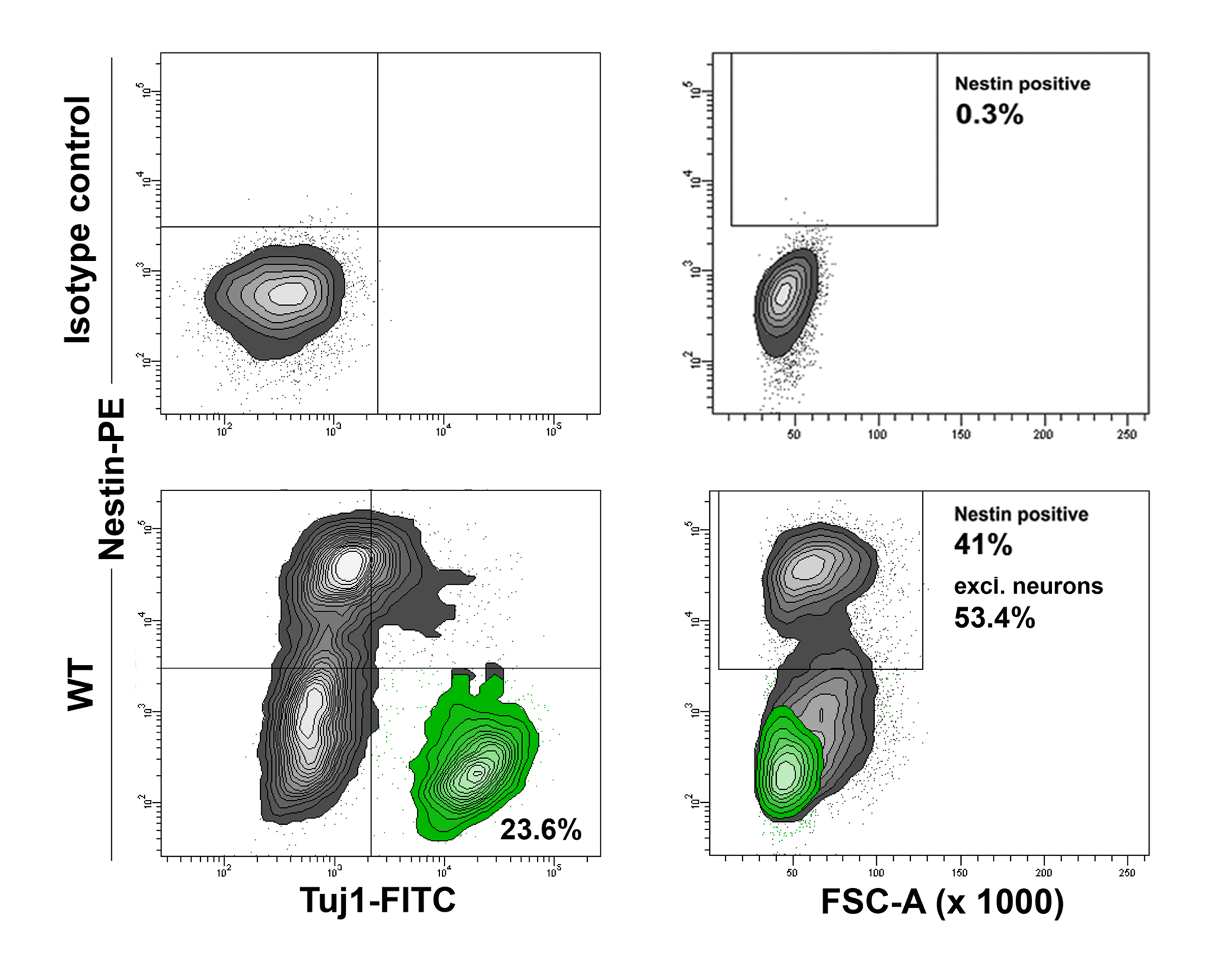

Supplement: Figure S4 — Supporting data to Figure 3. Flow cytometric analysis of wild type (WT) ESCs cells stained with isotype controls or Nestin and Tuj1 specific antibodies. The double staining allows the identification of Nestin positive neural progenitors and Nestin-negative/Tuj-positive mature neurons (highlighted in green). The left panel shows the Nestin-PE versus Tuj1 and the right panel indicate the Nestin-PE against forward scattering (FSC) of the same sample. Since Tcf3 over expressing clones gave rise to 0.1% mature neurons in average, the Nestin-PE versus FSC has been used in Figure 3. (TIF) [file pgen.1003424.s004.tif]

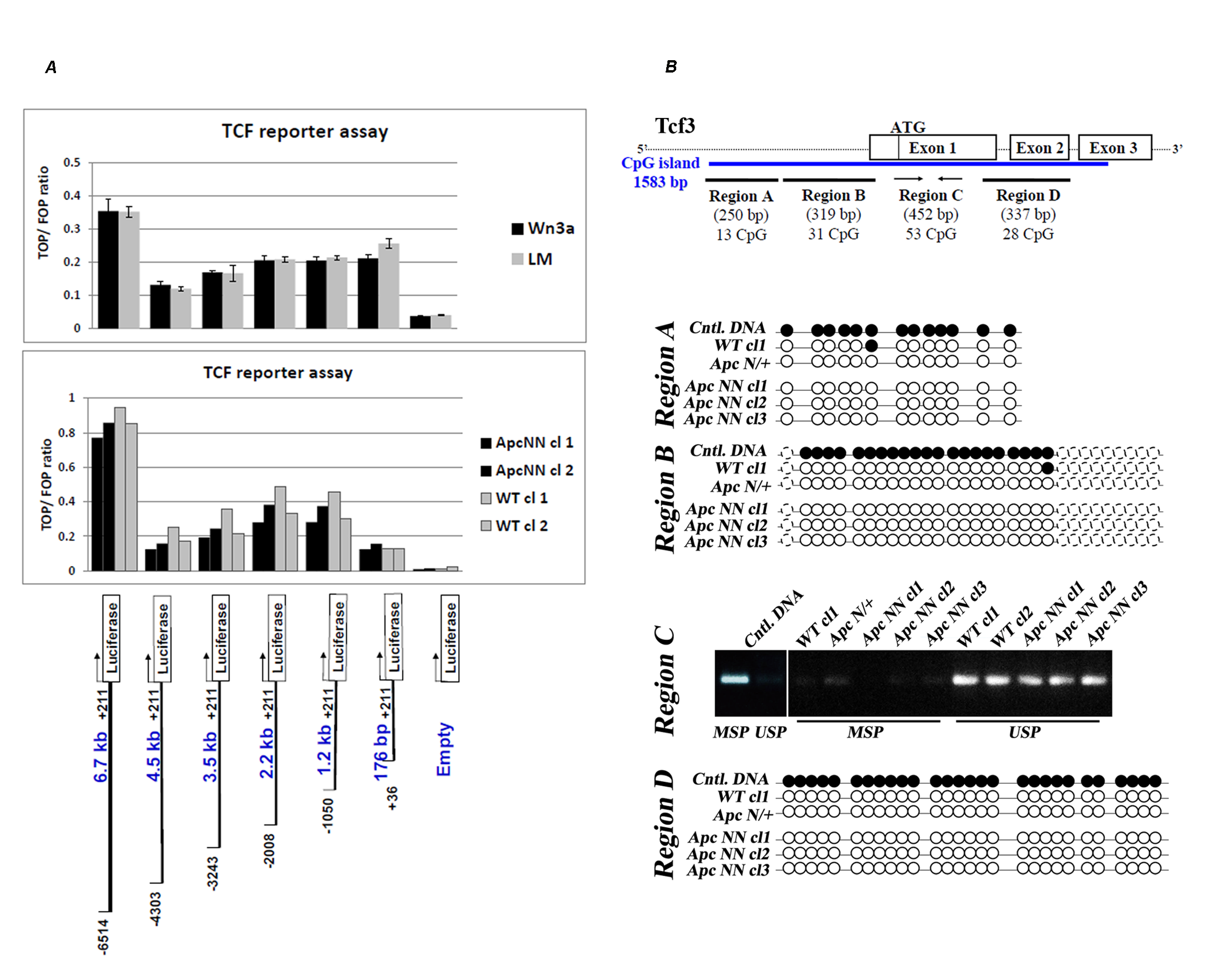

Supplement: Figure S5 — A. Tcf3 promoter activity in Wnt high and Wnt low ESCs. Luciferase constructs containing different Tcf3 promoter fragments were co-transfected with Renilla luciferase and the relative promoter activity is shown after normalization to Renilla-luciferase values. To monitor the effect of Wnt signaling on Tcf3 promoter activity, luciferase constructs were transfected in wild type ESCs, followed by 24 h treatment with Wnt3a-condition medium (Wnt3a) or L-control medium (LM). Similarly, luciferase constructs were transfected in 2 independent clones of ApcNN or wild type ESCs and promoter activity was measured after 48 h of transfection. The genomic location of different Tcf3 promoter fragments is depicted in the scheme. Bars represent n = 2±SD. B. DNA methylation analysis of Tcf3 promoter. Schematic representation of the mouse Tcf3 promoter defined by the 5′UTR and ∼2 kb large CpG island extending into exons 1–3. For the purpose of methylation analysis, the CpG island was subdivided into regions A, B, C and D. Genomic DNA was first bisulfite-converted and the individual regions either employed in bisulfite-specific PCR followed by DNA sequencing (region A, B and D), or used in methylation-specific PCR assays (region C). Arrows represent methylated (and un-methylated) specific primers for region C. PCR products from regions A, B and D were obtained from ApcNN, wild type and ApcN/+ (Apc1638N/+) ESCs and directly sequenced. ApcN/+ ESCs were employed as controls since they express similar Tcf3 levels compared to wild type ESCs (data not shown). The sequencing results are depicted as open and solid circles for unmethylated and methylated CpG dinucleotides, respectively. Dashed circles represent CpGs which were not included in the PCR products. Because of its extremely GC-rich sequence, PCR amplification of region C was carried out using methylated- and unmethylated-specific primers (MSP and USP) covering 4 different CpG dinucleotides. Control DNA (mouse genomic DNA where all Cp [file pgen.1003424.s005.tif]

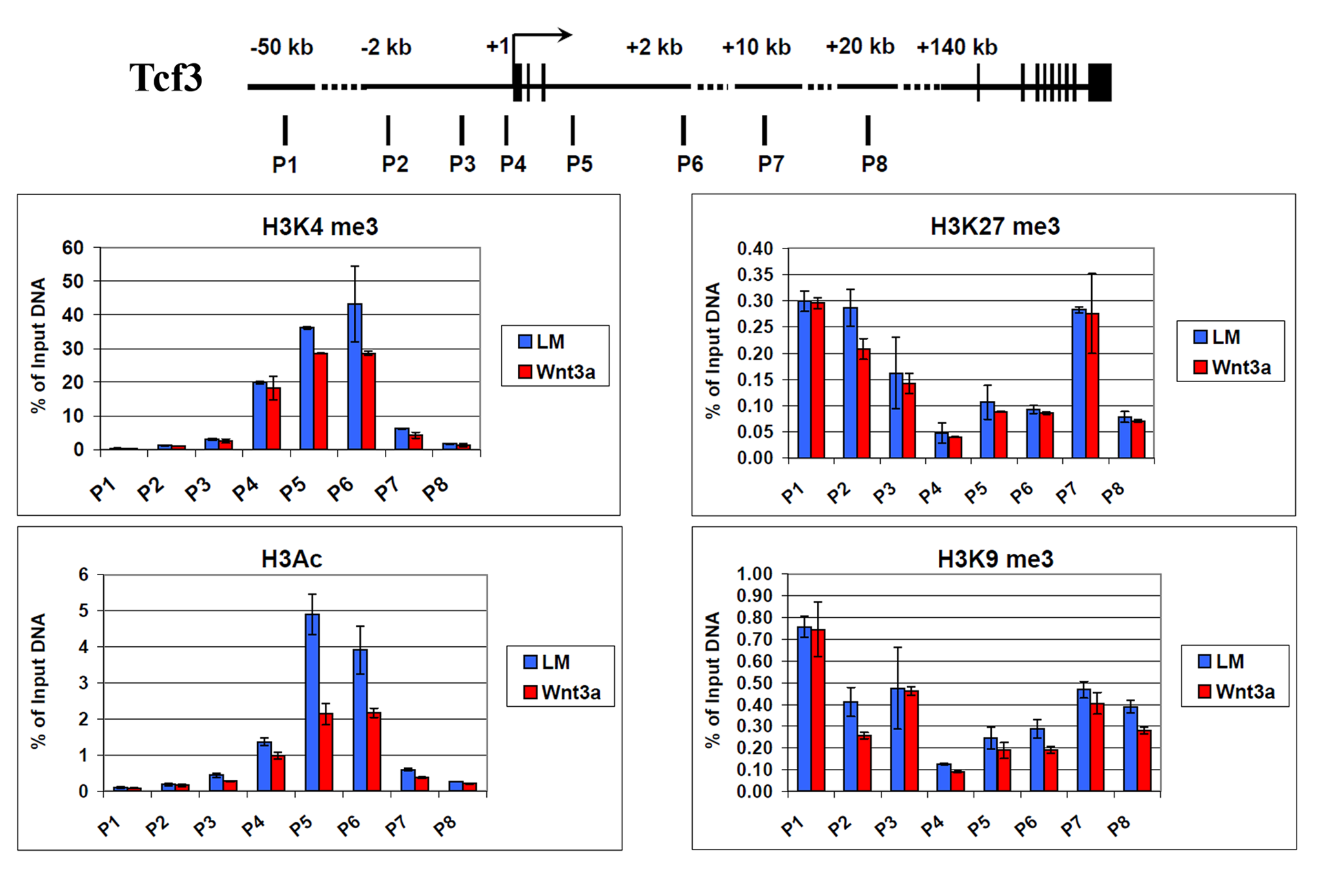

Supplement: Figure S6 — Transient activation of Wnt signaling in wild type ESCs reduces H3Ac and H3K4me3 activating marks in Tcf3 promoter. Bruce 4 wild type ESCs were cultured on gelatin-coated dishes and treated with Wnt3a condition medium or L-control medium for 12 h. Cells were used for ChIP-QPCR as described before. Values from each amplicon were normalized to input chromatin. Since no amplification was detected at the negative region (P1) from some of the immunoprecipitated chromatin, values are shown as percent of input DNA. Bars represent n = 2±SD. (TIF) [file pgen.1003424.s006.tif]

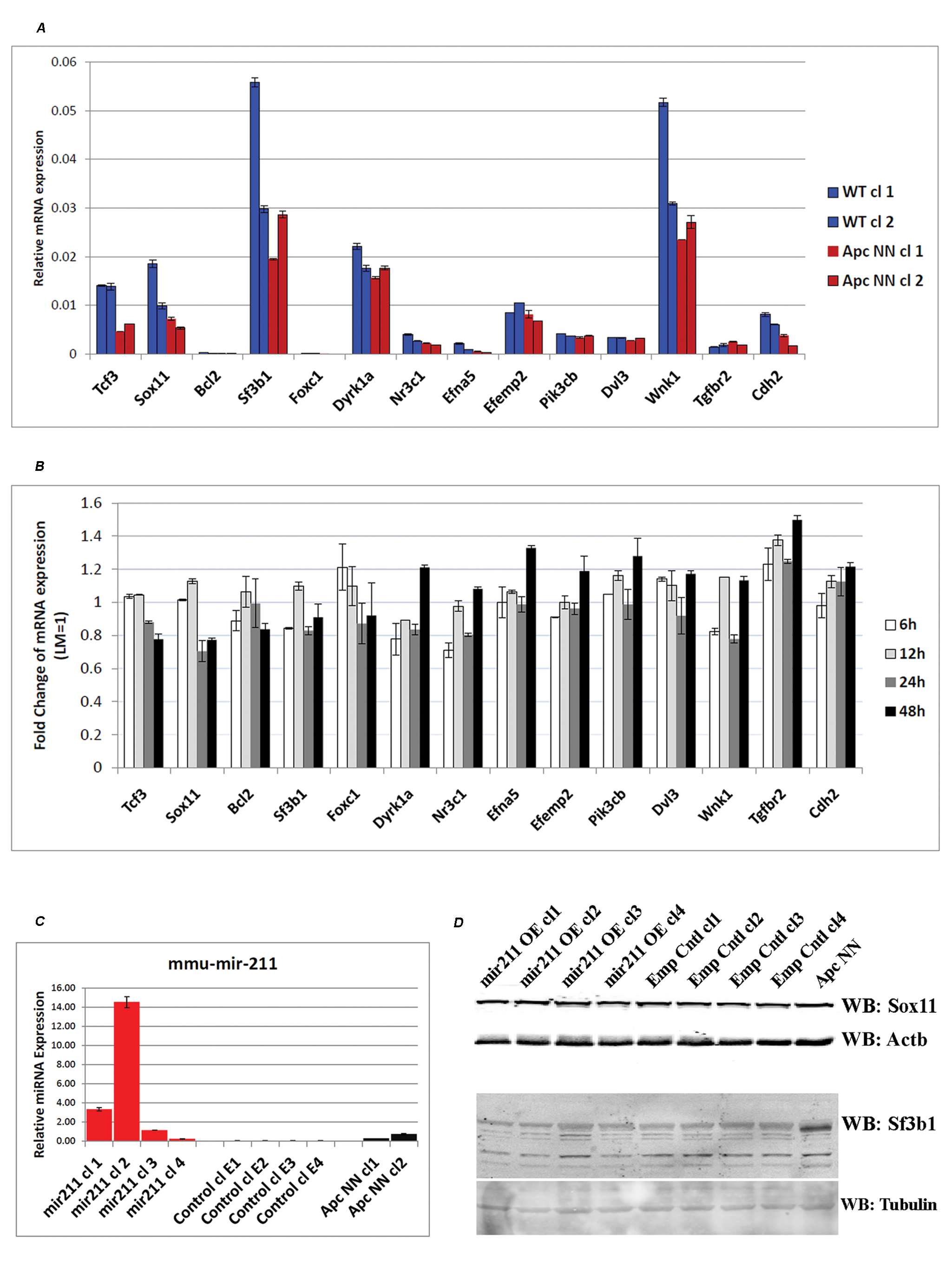

Supplement: Figure S7 — A. Histogram showing relative expression of selected miR-211 predicted targets in ApcNN and wild type ESCs. Two independent clones were used for each genotype. Actb was used for normalization. Bars represent n = 2±SD. B. Histogram showing relative expression of selected miR-211 predicted targets in wild type ESCs treated with Wnt3a condition medium or L-medium for different time intervals. The ratios of Wnt3a CM/L-medium are shown in the graphs. Bars represent n = 2±SD. C. qRT-PCR analysis of miR-211 expression in wild type ESCs stably expressing miR-211 or the corresponding empty vector. Two independent ApcNN ESC clones were included for comparison. snoRNA-234 was used for normalization.Bars represent n = 2±SD. D. Western blot analysis of the miR-211 predicted targets Sox11 and Sf3b1 in miR-211 over expressing cells and their wild type controls. (TIF) [file pgen.1003424.s007.tif]
